# Hyperbranched TEMPO-based polymers as catholytes for redox flow battery applications†

**DOI:** 10.1039/d4ra03925d

**Published:** 2024-10-18

**Authors:** Koosha Ehtiati, Ilya Anufriev, Christian Friebe, Ivan A. Volodin, Christian Stolze, Simon Muench, Grit Festag, Ivo Nischang, Martin D. Hager, Ulrich S. Schubert

**Affiliations:** a Laboratory of Organic and Macromolecular Chemistry (IOMC), Friedrich Schiller University Jena Humboldtstrasse 10 Jena 07743 Germany ulrich.schubert@uni-jena.de; b Center for Energy and Environmental Chemistry Jena (CEEC Jena), Friedrich Schiller University Jena Philosophenweg 7a Jena 07743 Germany; c Jena Center for Soft Matter (JCSM), Friedrich Schiller University Jena Philosophenweg 7 Jena 07743 Germany; d Helmholtz Institute for Polymers in Energy Applications Jena (HIPOLE Jena) Lessingstr. 12 – 14 07743 Jena Germany; e Helmholtz-Zentrum Berlin für Materialien und Energie GmbH (HZB) Hahn-Meitner-Platz 1 14109 Berlin Germany

## Abstract

Application of redox-active polymers (RAPs) in redox flow batteries (RFBs) can potentially reduce the stack cost through substitution of costly ion-exchange membranes by cheap size-exclusion membranes. However, intermolecular interactions of polymer molecules, *i.e.*, entanglements, particularly in concentrated solutions, result in relatively high electrolyte viscosities. Furthermore, the large size and limited mobility of polymers lead to slow diffusion and more sluggish heterogeneous electron transfer rates compared to quickly diffusing small molecules. Although a number of RAPs with varying electrolyte viscosities have been reported in the literature, the relation between the RAP structure and the hydrodynamic properties has not been thoroughly investigated. Herein, hyperbranched 2,2,6,6-tetramethylpiperidinyloxyl (TEMPO)-based polymers intended for application as low-viscosity catholytes for RFBs are presented and the influence of the structure and the molar mass distribution on the hydrodynamic properties is investigated. A new synthesis approach for TEMPO-based polymers is established based on step-growth polymerization of a TEMPO-containing monomer using an aza-Michael addition followed by a postpolymerization modification to improve solubility in aqueous solutions. The compact structure of hyperbranched polymers was demonstrated using size-exclusion chromatography (SEC) with viscometric detection and the optimum molar mass was found based on the results of viscometric and crossover investigations. The resulting RAP revealed a viscosity of around 21 mPas at a concentration corresponding to around 1 M TEMPO-containing units, according to the calculated mass of the repeating unit, showing potential for high capacity polymer-based catholytes for RFBs. Nevertheless, possible partial deactivation of TEMPO units lowered the active TEMPO concentration of the hyperbranched RAPs. A faster diffusion and higher charge transfer rate were observed for the hyperbranched polymer compared to the previously reported linear polymers. However, in RFB tests, a poor performance was observed, which is attributed to the side reactions of the oxidized TEMPO moieties. Finally, pathways for overcoming the main remaining challenges, *i.e.*, high loss of material during dialysis as an indication of being prone to crossover, the partial deactivation of TEMPO moieties, and the subsequent side reactions under battery conditions, are suggested.

## Abstract 2

Hyperbranched TEMPO-based polymers are investigated as catholytes for redox flow batteries revealing relatively low viscosity, rapid diffusion, and fast charge transfer. Remaining challenges are the polymers being prone to crossover and having a low cycling stability.

## Graphical abstract

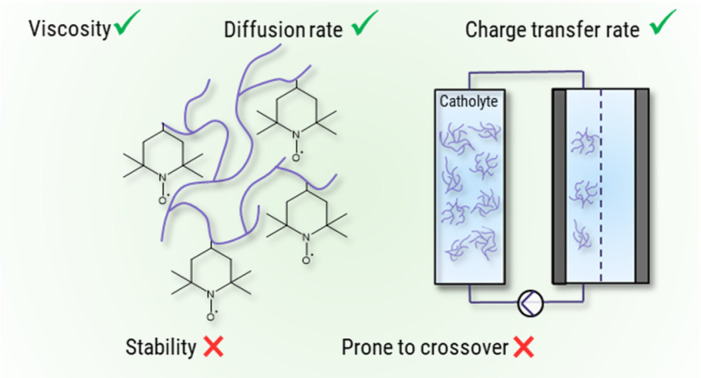

## Introduction

The increasing share of solar and wind power in the total energy supply requires long-duration energy storage to compensate the natural intermittency of these energy resources.^1^ Redox flow batteries (RFBs) provide a promising solution for long-duration energy storage possessing the advantage of decoupled scaling of the output power from the energy capacity. However, the cost targets for energy storage have not yet been met even for all-vanadium redox flow batteries which constitute the most technologically mature type of redox flow batteries.^2^ Cost analysis of such systems reveals that the cost of the cell stacks alone is higher than the price target for the overall system highlighting the importance of cost reduction for the cell stacks.^3^

One common approach to reduce the stack cost is to increase power density of individual cells, through reduction of the internal resistance and/or increasing the cell voltage.^4–6^

Alternatively, substitution of expensive cell parts has been examined. With regard to this, applying redox-active polymers (RAPs) instead of small molecules has been demonstrated to enable the substitution of expensive ion-exchange membranes by relatively inexpensive size-exclusion membranes *via* introduction of the size-exclusion mechanism for crossover prevention.^7–9^ The size-exclusion mechanism can also be achieved through application of micellar structures, slurries, and microgels.^10–13^

On the downside, concentrated polymer solutions are highly viscous at high concentrations and the use of lower polymer concentrations limits the battery energy efficiency and the electrolyte capacity.^7,8,14,15^ The first reported polymer-based RFB comprised a poly(2,2,6,6-tetramethylpiperidinyloxyl) (TEMPO)-based catholyte in which a TEMPO-containing monomer was copolymerized with a cationic comonomer to improve solubility in aqueous solutions.^8^ The applicable capacity of this polymer solution was 10 A h L^−1^ with a viscosity of 17 mPas. Later, Hagemann *et al.* and Fu and Zhang *et al.* were able to reduce the electrolyte viscosity *via* copolymerization with a zwitterionic comonomer, and terpolymerization using anionic comonomers, respectively.^7,14^ However, the relation between the polymer structure and hydrodynamic properties remains under-investigated.

The viscosity of polymer solutions is highly dependent on the polymer concentration, chain length, and salt concentration (in case of polyelectrolytes).^16–18^ Scaling relations for an uncharged polymer describes that while the specific viscosity scales with polymer concentration with scaling power of 1.25 in the unentangled semi-dilute regime, the scaling becomes much stronger after entanglements are formed with scaling power of 3.75 in the entangled semi-dilute regime. As a consequence, the viscosity of highly concentrated polymer solutions is strongly related to their ability to form entanglements. It is known that hyperbranched polymers possess a more compact structure compared to linear polymers at the same molar mass.^19–22^ As a result of such compact structure, hyperbranched polymers are less prone to form entanglements and, thus, enabling a weaker dependency of viscosity on the polymer concentration than that of linear polymers.^23–25^

We herein report hyperbranched TEMPO-based polymers as potentially low-viscosity catholytes for RFBs aiming to improve the electrolyte capacity. We employ an oxygen-tolerant synthesis of hyperbranched TEMPO-based polymers and thoroughly investigate the hydrodynamic properties of the resulting hyperbranched polymers with varying molar masses and concentrations. Furthermore, the electrochemical properties of the hyperbranched polymers are investigated and their potential application in RFBs is assessed.

## Experimental

### Materials

All starting materials were purchased from commercial sources, *i.e.*, Fisher Scientific, Tokyo Chemical Industry (TCI), and Sigma-Aldrich, and were used as received unless otherwise stated. 4-Amino-1-oxy-2,2,6,6-tetramethylpiperidine (4-amino TEMPO, 95%) was obtained from Apollo Scientific, and *N*,*N*′-dimethyl-4,4′-bipyridinium dichloride (MV) was obtained as a 45% w/w solution in water from JenaBatteries GmbH, Germany. Organic solvents were distilled before use and deionized water was utilized to prepare aqueous solutions. Two different dialysis membranes (Spectra/Por® Biotech CE MWCO 100–500 with width of 16 mm, and Spectrapor 6 MWCO 1,000, Spectrum Laboratories, Inc. with width of 45 mm) were used for dialysis.

### Synthesis of hyperbranched polymers using 4-amino TEMPO and 1,3,5-triacryloylhexahydro-1,3,5-triazine (triacrylamide) terminated with 1-methylpiperazine (HPT-I0 to HPT-I3)

In a typical polymerization batch, first 1,3,5-triacryloylhexahydro-1,3,5-triazine (6.232 g, 25 mmol) was placed in a 50 mL round bottom flask to which 12 mL methanol was added. 4-Amino TEMPO (4.282 g, 25 mmol) was dissolved in 6 mL methanol and was added to the flask at room temperature (RT). The reaction mixture was stirred at RT for 30 min. For the HPT-I1, HPT-I2, and HPT-I3 samples, at this point a trifunctional amine (1-(2-aminoethyl) piperazine, AEP) was added to increase the molar mass in molar ratios of 0.1 : 1, 0.2 : 1, and 0.3 : 1, relative to 4-amino TEMPO, respectively. After addition of AEP, the reaction mixture was stirred at RT for another 15 min. Next, the polymerization batches were placed in a preheated oil bath which was set at 40 °C. After 4 h of polymerization at 40 °C, the polymerizations were terminated by addition of another amine to consume all remaining double bonds again through an aza-Michael addition. In case of HPT-I0, HPT-I1, HPT-I2, and HPT-I3, the reaction was terminated by addition of excess amount of 1-methylpiperazine (5.5 mL, 50 mmol) and stirring for another 30 min at 40 °C. The polymer product was obtained by precipitation in diethyl ether. The samples that were characterized at neutral state were obtained after dissolution in water and subsequent freeze drying.

### Synthesis of hyperbranched polymers using 4-amino TEMPO and triacrylamide (1,3,5-triacryloylhexahydro-1,3,5-triazine) terminated with 4-amino TEMPO (HPT-II1)

In case of HPT-II1, the polymerization procedure was similar to HPT-I1 but the batch was four times larger and was prepared in a 250 mL round bottom flask aiming for sufficient material for electrochemical and battery tests. The main difference in this case was the termination which was performed using 4-amino TEMPO instead of 1-methylpiperazine to include more TEMPO units in the polymer chains and to further increase the capacity. To prevent possible crosslinking, 4-amino TEMPO was only added at RT to limit the reaction of the second hydrogen of 4-amino TEMPO for termination. In this case after 4 h of polymerization the reaction mixture was removed from the oil bath and was cooled to RT before adding the 4-amino TEMPO (17.126 g, 100 mmol) which was dissolved in 24 mL methanol. After 30 min of reaction, the product was obtained through precipitation in diethyl ether.

### Synthesis of hyperbranched polymers using cyclohexylamine as model system

To investigate the kinetics of the aza-Michael addition and its potential for polymerization, cyclohexylamine was used as a model system before testing 4-amino TEMPO. In a typical polymerization batch 1,3,5-triacryloylhexahydro-1,3,5-triazine (6.232 g, 25 mmol) was placed in a 50 mL round bottom flask to which 12 mL methanol and cyclohexylamine (2.479 g, 25 mmol) were added. The reaction mixture was stirred at RT for 5 min before placing in the preheated oil bath set at 40 °C for desired durations. The reaction was similarly terminated using an excess amount of 1-mehylpiperazine. Conversions were obtained based on ^1^H NMR measurements. An internal standard (benzaldehyde dimethylacetal) was added into the reaction mixture to facilitate data analysis by ^1^H NMR.

### Synthesis of the linear TEMPO-based polymer (PTMA)

In a round bottom flask, 2,2,6,6-tetramethyl-4-piperidyl methacrylate (22.53 g, 100 mmol) and 4,4′-azobis(4-cyanopentanoic acid) (350 mg, 1 mmol, 80%) were dissolved in a mixture of 80 mL methanol and 20 mL water and bubbled with argon for 45 min. The flask was subsequently placed in a preheated oil bath and stirred at 70 °C for 6 h. Subsequently, the reaction mixture was diluted with 100 mL methanol and cooled to RT. Na_2_WO_4_·2H_2_O (0.66 g, 2 mmol) were dissolved and 28.5 mL H_2_O_2_ (50% in water, approx. 5 eq.) were added dropwise. The mixture was heated to 40 °C and stirred for 65 h. The formed chunk of polymer was crushed into small pieces, separated on a POR 3 glass frit and washed with a methanol/water 2/1 mixture. The residue was dissolved in 100 mL acetone and precipitated in 1 L of cold methanol/water (10/1). The product was collected on a POR 3 glass frit, washed with a methanol/water mixture (2/1) and water, and dried under vacuum.

### Methylation of the hyperbranched polymers

Methylation was performed using an excess amount of iodomethane in methanol for three days. This harsh condition was used to maximize quaternization of tertiary amines of the polymer. For around each 1 g of polymer 7 mL of methanol and 2 mL of iodomethane (32 mmol) were used corresponding to around five times in excess. The product was collected through precipitation in diethyl ether. The remaining unreacted iodomethane was neutralized by addition of the diethyl ether solution to an aqueous ethanolamine solution until obtaining a final volumetric ratio of 10 : 30 : 60 for ethanolamine : diethyl ether : water.

### Ion exchange

The polymer product after methylation was dispersed in a 1 M NaCl aqueous solution (around 4 mL of aqueous solution was used for each 1 g of polymer) and was dialyzed against 1 M NaCl solution. Two different types of membranes were used with molecular weight cut off (MWCO) values of 100 to 500 Da (Spectra/Por® Biotech CE MWCO 100–500), and 1 000 Da (Spectrapor 6 MWCO 1 000). HPT-I0 to HPT-I3 were dialyzed against 1 M NaCl for two nights with renewal of the solution on the middle day. The polymer samples were not completely soluble at the beginning but as the ion exchange progressed, the samples turned into a clear orange solution. On the final day, the solution was replaced by deionized water to remove the excess salt and dialysis was continued for in total 6 h against deionized water where the deionized water was once renewed after the first 4 h.

Time limitation was due to the sensitivity of the 100 to 500 Da dialysis tubes which were already very swollen after 6 h of dialysis against deionized water. In the case of HPT-I0 which was dialyzed for around 15 minutes longer, a damage occurred to the top of one of the two dialysis tubes causing uncertainties with the amount of obtained material. For this practical limitation, only the 1 000 Da tube was chosen to dialyze the HPT-II1 sample. In this case after a round of dialysis against NaCl solution and deionized water, a trace amount of iodide was observed in the sample (detected by CV, for more details see the ESI S6†) and further dialysis against NaCl solution and deionized water was applied to completely remove the trace of iodide. The polymer products were finally obtained by freeze drying. The masses of the obtained polymer (data of Fig. 5a) were obtained as the ratio of (mass of obtained polymer)/(expected polymer mass with Cl^−^) where the expected polymer mass with Cl^−^ is calculated based on the mass of the polymer used before dialysis (with I^−^) and the iodide content of each sample (measured by elemental analysis).

Nuclear magnetic resonance (NMR) was performed on a Bruker Avance I (300 MHz) spectrometer. Elemental analysis (halogen analysis) was measured with a TLalpha20 (Si Analytics GmbH, Germany) titrator.^26^ UV absorption of the polymer samples were obtained using a PerkinElmer Lambda 750 UV-Vis spectrometer (1 cm quartz cuvette). The radical content of the TEMPO-based polymers was measured *via* electron paramagnetic resonance spectroscopy (EPR). X-band EPR spectra were acquired on an EMXmicro CW-EPR spectrometer from Bruker, Germany. The SpinCountQ software module was used for the determination of the spin count. A known PTMA polymer (radical content of 80%, determined through redox titration) was used as a reference. The radical contents of the TEMPO-containing compounds were determined from the mean values derived from the EPR spectra of three samples per compound.

### Size-exclusion chromatography (SEC) with viscometric detection

The molar mass distribution and the Kuhn–Mark–Houwink–Sakurada (KMHS) analysis were obtained using SEC with viscometric detection. The measurements were conducted on a Shimadzu 10er series instrument equipped with a SDV guard/Linear M (5 μm particle size) column (Polymer Standards Service, PSS, Germany). Tetrahydrofuran (THF) with added amine (1% v/v *N*,*N*-diethylethylenediamine, DEEDA) was used as the eluent at a flow rate of 1 mL min^−1^ at 30 °C. The amine was added to the eluent to screen the possible interactions between the polymer chains and the column material. The polymer samples were prepared with a concentration of 10 mg mL^−1^ (for hyperbranched polymer samples) and 2 mg mL^−1^ (for the linear PTMA sample). A multiangle laser light scattering (MALLS) detector (SLD 7100; PSS, Germany; *λ* = 660 nm) was used to determine the molar masses and a viscometer (ETA-2010; PSS, Germany) was applied to measure the viscosity of the respective elution slices from the disperse polymer populations. The refractive index increment (d*n*/d*c*) of the polymers in the corresponding eluent was measured using a refractometer (Optilab rEX, Wyatt, Germany). The refractive index increments of HPT-I0 and HPT-I3 were similarly obtained as 0.123 mL g^−1^ and, thus, the same value was assumed for HPT-I1 and HPT-I2. For PTMA, a refractive index increment of 0.1019 mL g^−1^ was obtained.

**Fig. 1:**
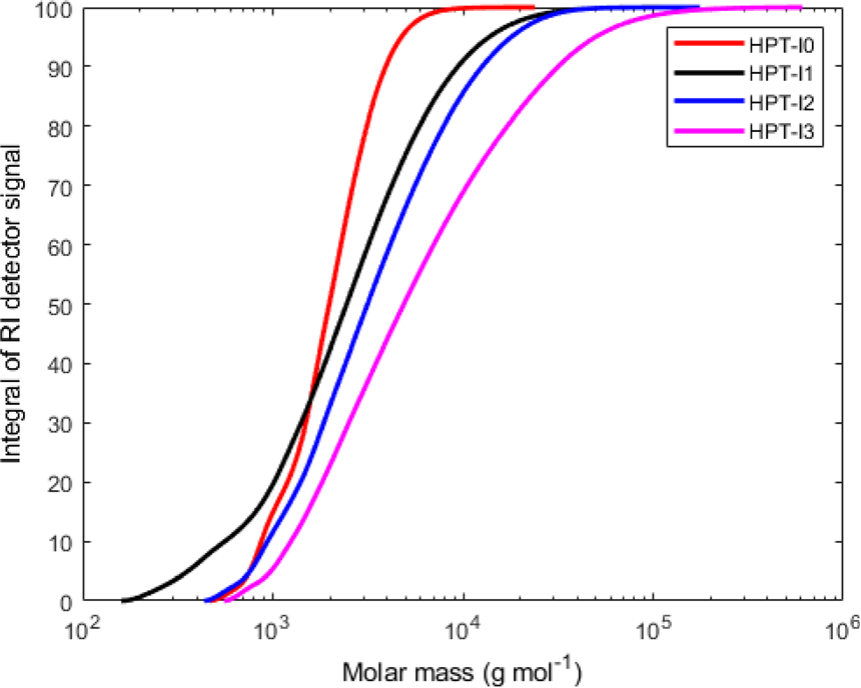
Cumulative molar mass distribution of the synthesized hyperbranched polymers based on SEC-MALLS.

**Fig. 2:**
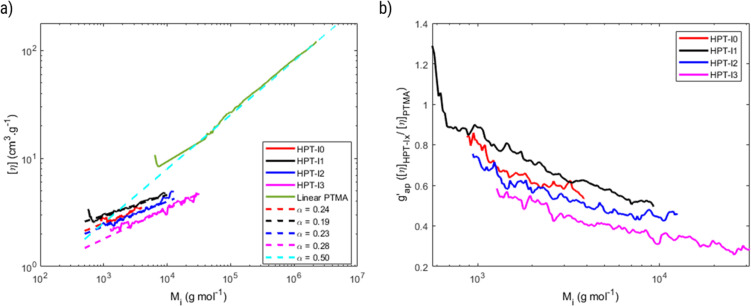
(a) Double logarithmic plot of the intrinsic viscosity [*η*] against the molar mass, *M*_i_, for the hyperbranched polymers and a linear TEMPO-based polymer analogue (PTMA) based on SEC with viscometric detection. (b) Apparent contraction factor, defined as the ratio of the intrinsic viscosity of the hyperbranched polymers to that of PTMA, as a function of molar mass.

The molar mass values reported in Fig. 1 and 2 are based on light scattering using the d*n*/d*c* and the instrument factor. The apparent contraction factor values were obtained as the ratio of the intrinsic viscosity of the hyperbranched polymers relative to the intrinsic viscosity of linear TEMPO-based polymer (PTMA) at the same molar mass:1
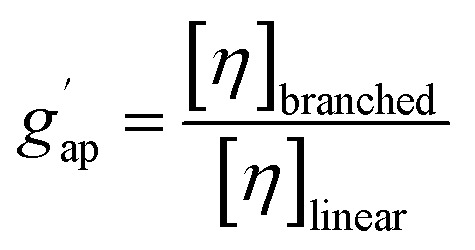
where the intrinsic viscosity of the linear polymer was calculated using the KMHS relation with the experimentally obtained values of *k* and *α*_KMHS_ for the linear PTMA (*k* = 0.147 and *α*_KMHS_ = 0.456). The results of SEC with viscometric detection presented in Table 1 are based on three measurements from which the average and the standard deviation were calculated. The results presented in Fig. 2 are the data of one representative test for each sample.

**Table 1:** Structural and hydrodynamic properties of the synthesized hyperbranched polymers

Polymer sample	AEP : TEMPO	*M*_w_ a (g mol^−1^)	[*η*]_g_^a^ (cm^3^ g^−1^)	*α*_KMHS_ a	*k* a	*M*_s,f_ b (g mol^−1^)	[*η*]^c^ (cm^3^ g^−1^)	*k*_H_ c	*v* d (cm^3^ g^−1^)
HPT-I0	0	3100 ± 700	3.2 ± 0.1	0.24 ± 0.05	0.55 ± 0.23	1100	4.8	1.7	0.74
HPT-I1	0.1 : 1	4100 ± 300	3.7 ± 0.1	0.19 ± 0.04	0.83 ± 0.24	1700	6.3	1.0	0.73
HPT-I2	0.2 : 1	7600 ± 900	3.9 ± 0.3	0.23 ± 0.05	0.57 ± 0.27	2900	6.1	1.5	0.72
HPT-I3	0.3 : 1	18 300 ± 2400	4.4 ± 0.2	0.28 ± 0.04	0.32 ± 0.09	11 300	10.2	0.9	0.73

a Measured using SEC with viscometric detection.

b Measured using AUC and based on the intrinsic viscosity values obtained from viscometry using capillary ball combination.

c Obtained from viscometry using capillary ball combination.

d Obtained from densimetry.

### Viscometry and densimetry

Dynamic and intrinsic viscosities of the polymer solutions were determined using a Lovis 2000 ME Microviscometer (Anton Paar, Graz, Austria) operating with the rolling ball principle. The system was equipped with glass capillaries (inner diameters 1.59 mm and 1.8 mm) and a gold ball with a diameter of 1.5 mm. For most polymer samples, a typical capillary inclination angle of 50° was chosen to perform the measurements. However, to ensure that the viscosity data corresponds to zero shear viscosity, the angular dependency was investigated for the polymer with the highest molar mass (see ESI S12† for more details). Experiments were performed at 20  °C. The viscosity values reported here are the average of at least six individual ball time measurements with the standard deviations being lower than 1%. The intrinsic viscosity, [*η*], of the polymer solutions was determined by procedures reported elsewhere.^27^

A density meter DMA 4500 M (Anton Paar, Graz, Austria) was used for the density measurements of the polymer solutions at 20 °C. The partial specific volume of the polymers was determined according to the procedure described elsewhere.^28^

### Analytical ultracentrifugation (AUC)

Sedimentation velocity analytical ultracentrifugation (AUC) experiments were performed with a ProteomeLab XL-I analytical ultracentrifuge (Beckman Coulter, Brea, CA). The cells were equipped with double-sector aluminum centerpieces (12 mm optical path) and sapphire windows. The cells were filled with *ca.* 440 μL of pure solvent in the reference sector and *ca.* 420 μL of the polymer solutions in the sample sector (at a concentration of 1 mg mL^−1^). Before the measurements, cells were inserted into an eight-hole rotor (An-50Ti) and kept in the centrifuge chamber at 20 °C for 2 h. The AUC runs were performed at a rotor speed of *ω* = 50 000 rpm using the interference optical detection system with scans acquired at a time interval of 90 s. Sedimentation-diffusion analysis was performed with SEDFIT (version 16.1c) by using sedimentation-diffusion analysis with the *c*(*s*) model.^29^ The molar masses, *M*_s,f_, were calculated by the modified Svedberg equation, 
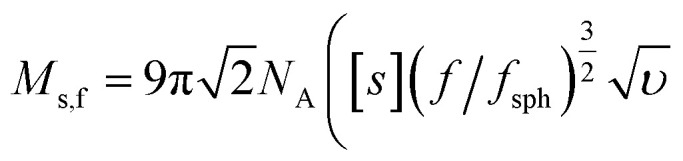
, where *N*_A_ is Avogadro number, 
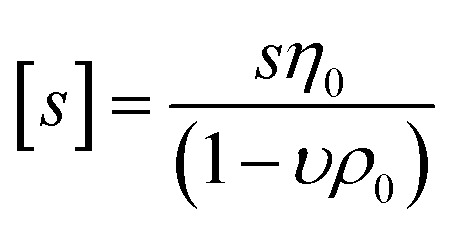
 is the intrinsic sedimentation coefficient, *η*_0_ and *ρ*_0_ are solvent viscosity and density respectively, *υ* is partial specific volume, and *f*/*f*_sph_ is the translational frictional ratio.

### Cyclic voltammetry and chronoamperometry

Cyclic voltammetry (CV) and chronoamperometry (CA) were carried out using a VMP-300 multichannel potentiostat (BioLogic, France) on polymer solutions with concentrations equivalent to 5.7 mM TEMPO in 1.5 M NaCl aqueous solution. The pH value of the solutions was adjusted to 2.0 to protonate any remaining unquaternized tertiary amine groups. A three-electrode setup was used with a glassy carbon working electrode (disc with a diameter of 1.6 mm), a coiled platinum wire counter electrode (diameter of 0.25 mm) and an Ag/AgCl (3 M NaCl) reference electrode.

CV was conducted by scanning from 0.2 V up to 1.1 V *versus* the reference electrode for six consecutive cycles with scanning rates from 5 mV s^−1^ up to 1 000 mV s^−1^. Voltammetry experiments were conducted with a random order of scan rates to avoid systematic errors of electrode surface changes on the scan rate dependency and the solution was stirred shortly between the tests to ensure the same boundary conditions for every test. Determination of the peak-to-peak potential and the peak current was based on the first cycle. To determine the diffusion coefficient, the range of scan rates was selected where the peak-to-peak potential was independent of the scan rate. The diffusion coefficient for a one-electron reaction was obtained based on the eqn (2):^30^2
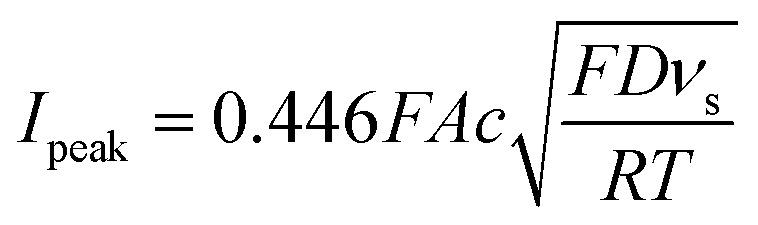
where *F* is the Faraday constant, *A* is the electrode surface area, *c* is the bulk concentration of the redox-active moiety, *D* is the diffusion coefficient, *ν*_s_ is the scan rate, *R* is the universal gas constant, and *T* is the temperature.

CA was conducted by applying a step potential of 1.0 V for 60 s. Before application of this step potential the electrode was held at the potential of 0.2 V for 60 s. Another period of 60 s at 0.2 V was applied after the step potential to ensure all the oxidized molecules were reduced again so the bulk concentration remains constant. The test was conducted three times, and the solution was shortly stirred in between. The diffusion coefficient was calculated based on the Cottrell equation for a semi-infinite diffusion:^30^3
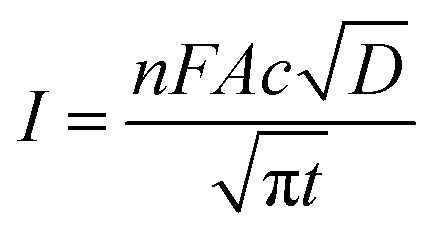
where *n* is the number of transferred electrons, and *t* is the time.

### Rotating disc electrode voltammetry

Rotating disc electrode voltammetry was conducted using a multichannel potentiostat (BioLogic, France) on a polymer solution equivalent to 2.0 mM TEMPO in 1.5 M NaCl at pH = 2.0. A three-electrode setup was used with a 5 mm diameter disc glassy carbon working electrode, a platinum wire counter electrode and an Ag/AgCl reference electrode. At each rotation speed, CV was conducted between 0.2 V and 1.1 V *versus* the reference electrode for three consecutive cycles at the scan rate of 5 mV s^−1^. First, a CV was conducted at 0 rpm from which the standard potential was obtained. Then CVs were conducted at various rotation speeds starting from 100 rpm and up to 2 500 rpm. Similarly, voltammograms of the background solution (1.5 M NaCl) were obtained at similar rotation speeds. The current data of the background solution was subtracted from the raw data of the polymer solution. The diffusion coefficient was obtained from the relation between the limiting currents (current at potential of 1.1 V) and the rotation speed as described in the Levich equation:^30^4
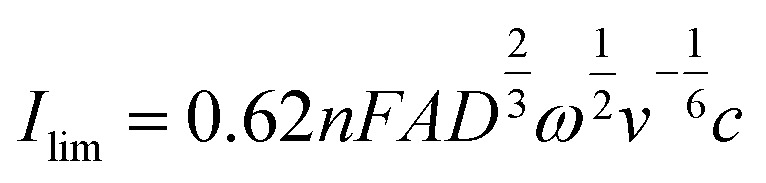
where *ω* is the rotation speed, and *v* is the kinematic viscosity. The value of kinematic viscosity was calculated based on the viscosity and density of a 1.5 M NaCl solution at 20 °C (
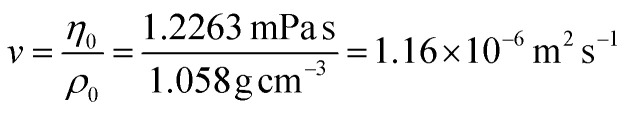
). The mass-transfer-independent kinetic current (*I*_k_) was obtained *via* the Koutecký–Levich equation:^31^5
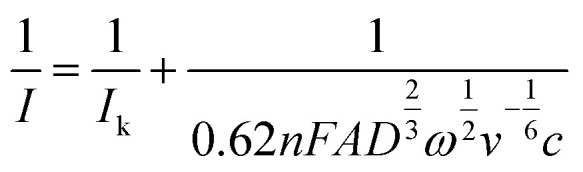


The kinetic currents were then plotted as a function of overpotential (here defined as the difference between the potential and the formal potential) to determine the heterogenous charge transfer constant (*k*^0^) and the transfer coefficient (*α*) as described for a one-step one-electron reaction:6
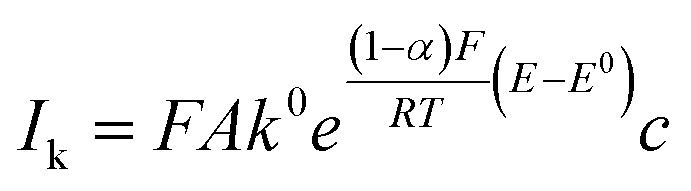
where *E*^0^ is the formal potential which here is determined as the average of the potentials of the anodic and cathodic peaks at rotation speed of 0 rpm.

### Redox flow battery experiment

A redox flow test cell (JenaBatteries GmbH, Germany) with a membrane area of 5 cm^2^ was used for the battery test. The cell was equipped with an anion-exchange membrane (fumasep FAA-3-50, Fumatech, Germany), graphite felt electrodes (GFA-6, SGL SE, Germany) and graphite composite current collectors (Mersen Deutschland Suhl GmbH, Germany). 10 mL of HPT-II1Cl solution in 1.5 M NaCl at pH = 2.0 with a concentration equivalent to 18.65 mM active TEMPO moiety was used as the catholyte corresponding to an overall capacity of 5 mAh. Around 25 mL of MV with the same concentration in the same supporting electrolyte was used as the anolyte. Both solutions were bubbled with argon to remove oxygen and subsequently transferred to a glovebox where the flow battery test was conducted. Galvanostatic cycling was applied at a constant current of 5 mA with potential limits of 1.0 V and 1.5 V. A peristaltic pump (Hei-FLOW Advantage, Heidolph, Germany) was used to control the flow of electrolytes.

## Results and discussion

### Synthesis of the hyperbranched polymers

**Scheme 1:**
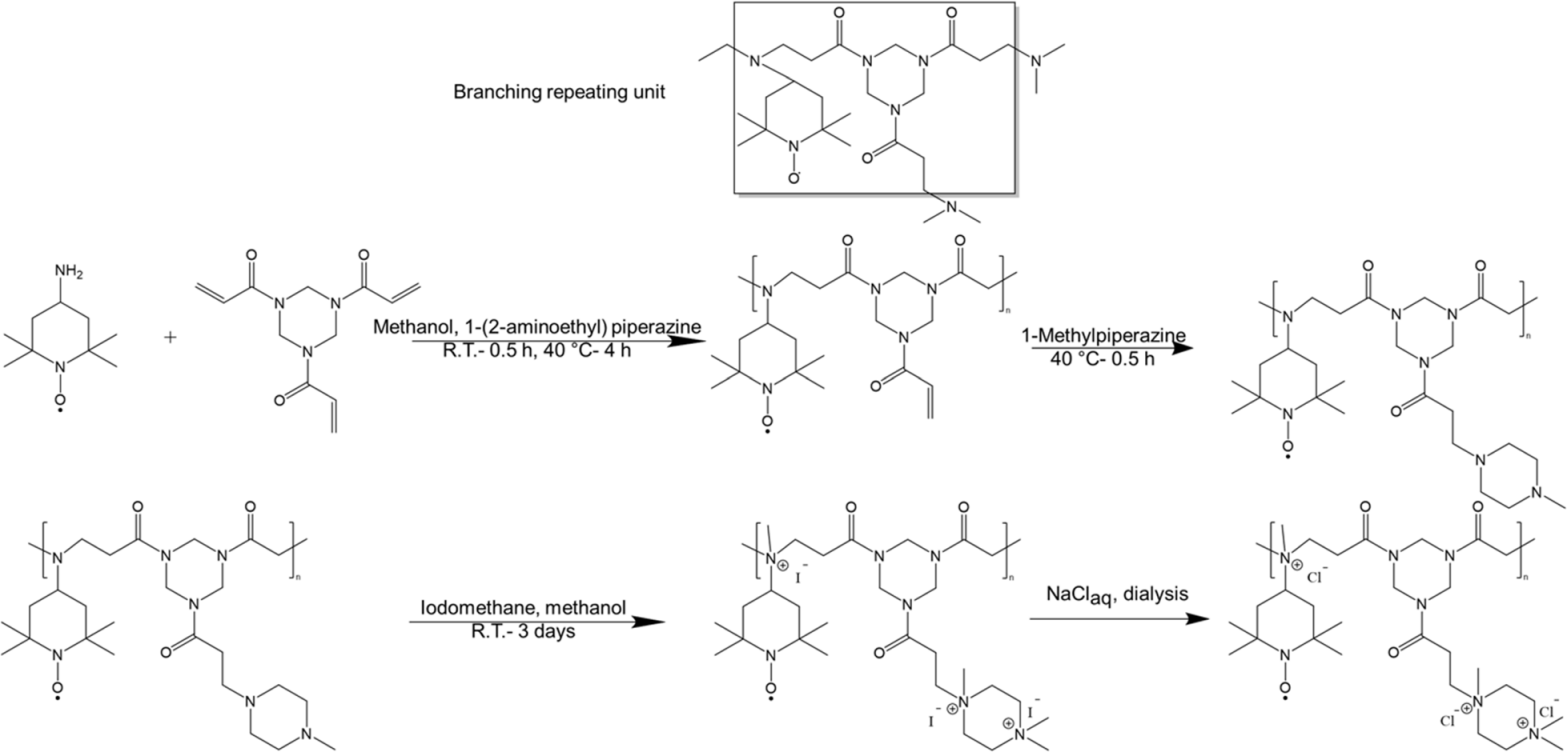
Schematic representation of the polymerization of 4-amino TEMPO and 1,3,5-triacryloylhexahydro-1,3,5-triazine and termination (first row). Post-polymerization modification of polymers through amine quaternization and ion exchange (second row).

Among various approaches to synthesize hyperbranched polymers, step-growth polymerization of monomers of difunctional (A_2_) and trifunctional (B_3_) is widely employed.^19,20,22,32^ Furthermore, groups of various reactivities, *i.e.*, A′A and B′B_2_ where the reaction between A′ and B′ is faster than that of between A and B, represents an effective approach to prevent gelation due to *in situ* formation and self-polymerization of AB_2_ molecules.^33–35^ Here we have synthesized hyperbranched TEMPO-based polymers using 4-amino TEMPO, as A_2_ monomer possessing a primary amine, which can react twice, and a trifunctional enoate, *e.g.*, triacrylate and triacrylamide, as B_3_ monomer possessing three reactive double bonds, through aza-Michael addition (Scheme 1). In an aza-Michael addition, primary amines are more reactive than the formed secondary amines resembling an A′A + B_3_ chemistry. Such a behavior helps preventing gelation through formation of AB_2_ molecules.^36,37^

As a model system, cyclohexylamine was first employed instead of 4-amino TEMPO, due to its availability and facility of NMR analysis compared to 4-amino TEMPO, to study the polymerization kinetics and molar mass variation over time. Methanol was used as a protic solvent to increase the rate of aza-Michael addition. Since acrylates are more reactive than acrylamides in aza-Michael addition, first a triacrylate (trimethylolpropane triacrylate) was used as B_3_.

Around 50% conversion of NH groups was obtained quickly but the reaction slowly progressed further reaching a conversion of around 90% after around 50 h corresponding to a theoretical degree of polymerization of only five (ESI S1†). This is in line with the higher reactivity of the primary amines compared to formed secondary amines in an aza-Michael addition. Longer polymer chains were aimed by having polymerization times of around four days which resulted in a conversion of 97% corresponding to a degree of polymerization around 16. However, transesterification of ester groups and subsequent shortening of polymer chains were observed as a drawback of ester groups in the backbone (see ESI S2†).

As an alternative, a triacrylamide (1,3,5-triacryloylhexahydro-1,3,5-triazine) was employed, which is expected to be more stable in polymerization reaction and less prone to hydrolysis under conditions of the final application compared to acrylates. In case of triacrylamide the reaction mixture was slightly heated to 40 °C to improve the solubility of the reactants and to increase the reaction rate of the formed secondary amines. In such conditions, around 90% conversion of the NH groups was already obtained after around 1 h of reaction (ESI S3†). Following the kinetics of the reaction, the initial polymers were obtained by variation of the polymerization time between around 4 to around 8 h (ESI S4†). At certain times, polymerization was terminated by addition of an excess amount of 1-methylpiperazine to consume all the remaining double bonds through aza-Michael addition (Scheme 1). With increasing polymerization time, the molar mass distribution became broader with an increasing fraction of long chains (*M*_w_ > 40 000 g mol^−1^). This behavior is expected for an A_2_ + B_3_ chemistry because longer chains, possessing higher number of functionalities, have higher probability for further growth compared to shorter chains leading to broadening of the molar mass distribution as the polymerization progresses. Since very long chains can potentially increase the viscosity of the polymer solutions which is undesirable, a short polymerization time of 4 h was chosen for the TEMPO-based samples in this study.

Polymerization of 4-amino TEMPO with 1,3,5-triacryloylhexahydro-1,3,5-triazine was performed under similar conditions. However, in this case molar masses of the obtained polymers were lower with narrower distributions compared to the cyclohexylamine polymerization (ESI S4†). Further attempts to increase molar mass by varying the stoichiometry, temperature, and time did not lead to an increase indicating that the molar mass was limited by the monomer functionality. To this end, various amounts of a trifunctional amine (AEP) were added to increase the overall amine functionality and, thus, to increase the molar mass. Fig. 1 and Table 1 show the properties of the polymers obtained by this strategy. The incorporation of both monomers and the chemical structure was supported by ^1^H NMR spectroscopy and UV-Vis absorption spectroscopy (ESI S5†).

After the polymerization, the polymers were further modified through methylation using iodomethane, as shown in Scheme 1 followed by ion exchange to substitute I^−^ by Cl^−^. Methylation and subsequent ion exchange were performed to improve the solubility of the redox-active polymers in aqueous solutions and to avoid any interference with the redox activity of the TEMPO moiety as tertiary amines and I^−^ ions can be oxidized within the potential window of TEMPO oxidation (see ESI S6† for more details). The methylation reaction was verified qualitatively by comparing the ^1^H NMR spectra of the polymers before and after amine quaternization and a high conversion (>90%) was quantitatively obtained using elemental analysis and acid titration (see ESI S7† for more details). Finally, the exchange of counterions from I^−^ to Cl^−^ was confirmed by cyclic voltammetry.

### Structural and hydrodynamic characteristics of the hyperbranched polymers: molar mass distribution and compactness

Hyperbranched polymers possess more compact structures compared to their linear counterpart.^23–25^ As a result of such compact structure, the impact on the viscosity of the solution is significantly reduced when compared to linear chains at the same molar mass. This is reflected in the lower intrinsic viscosity of hyperbranched polymers compared to their linear counterparts. To investigate the structure of synthesized polymers and make comparisons with a linear TEMPO-based polymer, the synthesized polymers were characterized by SEC with viscometric detection in THF with 1% v/v added *N*,*N*-diethylethylenediamine as the solvent (Fig. 1, 2 and Table 1). As can be seen in Fig. 1, while HPT-I0 shows a relatively low molar mass and narrow molar mass distribution, the molar mass increases and the molar mass distribution becomes broader as the amount of the trifunctional amine (AEP) increases from 0.1 : 1 to 0.3 : 1 for HPT-I1 to HPT-I3, respectively. This is expected because the presence of a trifunctional amine increases the probability for combination of two or three individual propagating chains. This effect can ultimately result in crosslinking at high amount of trifunctional amine. However, as discussed earlier, because 4-amino TEMPO shows an apparent lower functionality than expected for two functional groups in an aza-Michael addition, the addition of trifunctional amine in this case compensates for the lower functionality of 4-amino TEMPO and, thus, it does not result in crosslinking up to a ratio of 0.3 : 1.

Using SEC coupled with viscometric detection enabled estimation of the intrinsic viscosity, [*η*], of each molar mass elution slice, *M*_i_, of the disperse polymer population. The relationship of [*η*] and *M*_i_ can be described by an exponential Kuhn–Mark–Houwink–Sakurada (KMHS) scaling relationship, 
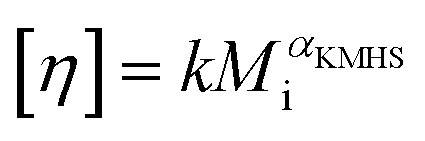
. A double logarithmic plot of [*η*] against *M*_i_ from SEC with viscometric detection enables the estimation of the exponent *α*_KMHS_. Generally, for linear polymer systems it assumes a value of 0.5 or larger. For ideal hyperbranched systems [*η*] is independent of the molar mass.^38^ Practically, for branched or hyperbranched systems, *α*_KMHS_ assumes values below 0.5.^39,40^

The acquired viscosity data of the hyperbranched polymers appear noisy, as observed in Fig. 2. This can be attributed to the compact nature of the hyperbranched polymers, low molar mass, relatively broad distribution, and overall low [*η*]-values. From the molar mass distribution of polymer samples, the middle 80% fraction of each sample is selected for investigating the KMHS relation. The resulting values of the exponent for the newly synthesized polymers determined over a limited molar mass range of around one order of magnitude are in the range of 0.19 to 0.28, indicating more compact polymer architectures compared to the linear polymer. Furthermore, the apparent contraction factor is obtained from the ratio of the intrinsic viscosity of the hyperbranched to that of the linear polymer at the same molar masses (calculated based on the obtained KMHS relation for linear PTMA), as shown in Fig. 2b.^19,40^ The contraction factor reflects the compactness of the hyperbranched polymers compared to linear chains with the same chemical structures.^19,41^ Here, due to synthesis and solubility limitations, a linear polymer with the same chemical structure was not synthesized and linear PTMA, containing TEMPO moieties but possessing a different backbone than that of the hyperbranched polymers, was used to compare the intrinsic viscosities. Hence, we have used the term “apparent contraction factor” to distinguish the ratio of intrinsic viscosities in our case from the contraction factor, which should ideally be obtained from polymers with the same chemical structure. For a wide range of molar masses, from around 1 000 to 30 000 g mol^−1^, the apparent contraction factor of all the polymer samples is between 0.8 to 0.3 and decreases with increasing molar mass, indicating the compactness of the hyperbranched polymers as compared to PTMA with similar molar mass. The much more compact structure of the hyperbranched polymers manifests itself at larger molar masses; at small molar masses the [*η*] values tend to converge such that there is no particular benefit in the low molar mass region in terms of intrinsic viscosity.

While SEC with viscometric detection could verify the more compact structure of polymers, although over a small and limited molar mass range, there are technical issues associated with the reported molar mass distribution. First, the intensity of the light scattering signal is low even at a rather high concentration of polymer samples used here (10 mg mL^−1^) which is attributed to the low molar mass of the polymer samples and the low value of refractive index increment. Additionally, the functional groups from the polymer backbone can interact with the column material interfering with the size-exclusion mechanism for separation of various polymer fractions. Such effect has previously been observed for branched polymers with functional groups with larger molecules being more likely to interact with the column.^42^ Here we have added 1% v/v of DEEDA to the eluent to screen such interactions. While addition of the amine enables proper elution of the sample, the polymer–column interactions cannot be completely excluded. We utilized another hydrodynamic technique known as analytical ultracentrifugation (AUC) to independently determine the molar masses, *M*_s,f_. We conducted sedimentation velocity experiments and sedimentation-diffusion analysis (Table 1 and S3†). More details can be found in the ESI S8.† The resulting values of the molar masses, *M*_s,f_, of the individual polymer populations are smaller than those from SEC-MALLS, an aspect that aligns with observations made with nonrelated polymer populations and when comparing SEC-MALLS and AUC.^43^ The trend of molar masses based on AUC follows the amount of AEP added in the polymerization. It is also confirmed that by addition of AEP, the polymer samples become more disperse. While the majority of chains are still small, larger chains are formed by addition of AEP.

### Hydrodynamic characteristics of the hyperbranched polymers in the neutral state: viscosity and density measurements

**Fig. 3:**
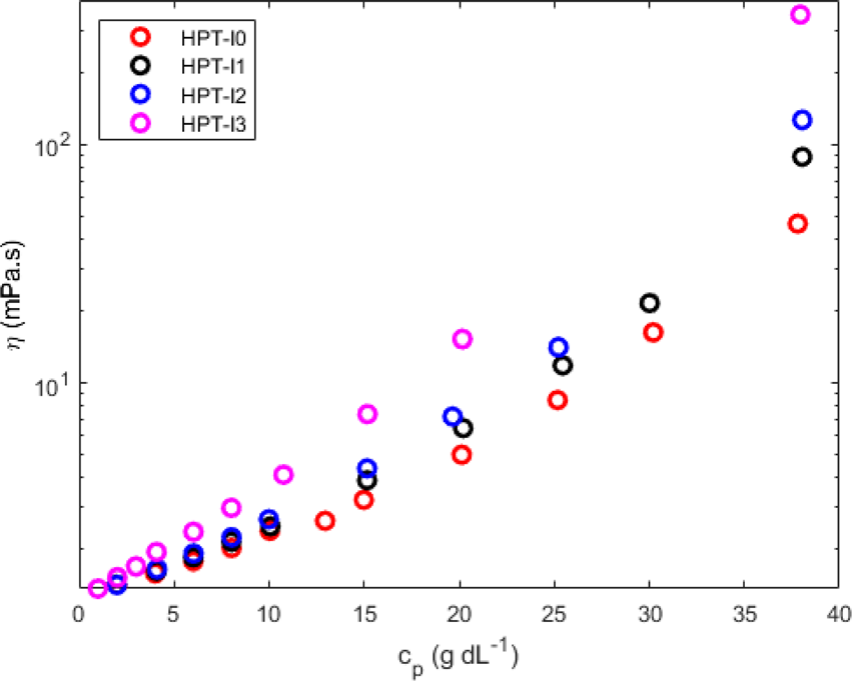
Dynamic solution viscosity of the synthesized hyperbranched polymers as a function of polymer concentration in ethanol measured with a capillary ball combination.

The difference in molecular properties as well affects the global solution viscosity, which was investigated over a wide range of concentrations (Fig. 3). To study the influence of molar mass on the viscosity of hyperbranched TEMPO-based polymers, the measurements were conducted on the polymer solutions in the neutral state (before methylation) in a nonaqueous solvent (ethanol) to exclude the influence of the polyelectrolyte effects. Concentration dependences of the dynamic viscosity of all newly synthesized polymers are shown in Fig. 3. Overall the dynamic viscosity of polymer solutions grows with increasing concentration. While at very low polymer concentrations, the viscosities of HPT-I0 to HPT-I3 have an insignificant difference, the effect of molar mass (Table 1) on the viscosity becomes more pronounced as the concentration increases leading to an about 8-fold difference between HPT-I0 (46 mPas) and HPT-I3 (348 mPas) at the highest mass concentration measured here (38 g dL^−1^).

**Fig. 4:**
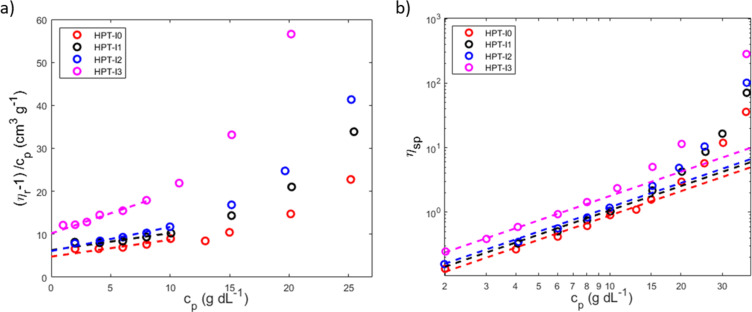
(a) Reduced viscosity of the hyperbranched polymer solutions as a function of polymer concentration and (b) double logarithmic plot of the specific viscosity as a function of polymer concentration obtained based on viscosity measurements of polymer solutions in ethanol using the capillary ball combination.

To investigate the influence of the molar mass on the intrinsic viscosity and the entanglement formation, reduced viscosity (
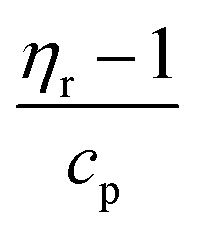
) and specific viscosity (*η*_sp_ = *η*_r_ − 1), where *η*_r_ is the ratio of the solution viscosity to the solvent viscosity known as relative viscosity and *c*_p_ is the polymer concentration, are plotted in Fig. 4. The Huggins extrapolations were performed to determine the values of intrinsic viscosity, [*η*], in a range of relative viscosities of *η*_*r*_ = 1.2–2.5, which corresponds to the dilute solution regime (Fig. 4a). The Huggins parameters for HPT-I0 to HPT-I3 are in the range of 0.9 to 1.7, which corresponds to previously observed values for hyperbranched polymer systems.^38^

Overall, the intrinsic viscosity values appear to be higher compared to those obtained from SEC with viscometric detection. This difference is attributed to the way those values are obtained. The global intrinsic viscosity obtained from SEC with viscometric detection is an average value of the intrinsic viscosity of polymer fractions of various molar masses, while viscometry is conducted on the entire sample solution in which the larger macromolecules have a larger influence on the viscosity than the smaller ones.

Comparing the highest and the lowest molar mass samples, it is observed that while the molar mass has increased with a factor of 6 or 10 (derived from SEC results and AUC results, respectively), the intrinsic viscosity has increased with a factor of around 2. This moderate dependency of the intrinsic viscosity on the molar mass is expected for hyperbranched polymer solutions as was discussed in the previous section with respect to the KMHS analysis. By plotting the specific viscosity as a function of polymer concentration in a double logarithmic scale, as shown in Fig. 4b, information about entanglement concentration of the polymer solutions is obtained. The dashed lines show a slope of 1.25 corresponding to the description of scaling relations for unentangled semi-dilute solutions. A positive deviation from this slope can be considered as an indication of the entanglement formation as the scaling power is higher in the entangled semi-dilute solution. While such deviation has already started from the concentration of around 11 g dL^−1^ for HPT-I3, the specific viscosities of HPT-I1 and HPT-I2 start to deviate at the concentration of around 15 g dL^−1^ and HPT-I0 shows such deviation starting from 20 g dL^−1^. Alternatively, the degree of dilution of a polymer solution is the product *c*[*η*], also known as the Debye parameter.^44^ This parameter describes the volume of the solution that is occupied by the polymer. Values of *c*_p_[*η*] < 1 indicate that the polymers occupy a smaller volume than the volume of the solution. A value of *c*_p_[*η*] of 1 is the polymer concentration at which the polymer occupies the entire solution volume. Apparently, this will result in similar concentration values as obtained from the double-logarithmic plots shown in Fig. 4b, not requiring knowledge of [*η*]. Values of *c*_p_[*η*] = 1 are found for polymer HPT-I3 being *c*_p_ = 9 g dL^−1^, for HPT-I2 and HPT-I1 *c*_p_ = 16 g dL^−1^, and for HPT-I0 *c*_p_ = 21 g dL^−1^. Those values appear to correlate with the ones above and derived from the double logarithmic plots in Fig. 4b.

Overall, it can be concluded that increasing the molar mass only moderately influences the intrinsic viscosity of hyperbranched polymers due to their compact structure. The viscosity of highly concentrated solutions is, however, still significantly affected by the molar mass. This can be due to a not ideally hyperbranched molecular architecture and the higher chance of entanglement formation for larger macromolecules resulting in a significant influence of the molar mass on the overall viscosity of entangled semi-dilute solutions.

### Viscosity-crossover trade-off: finding the optimum molar mass regarding viscosity and crossover

Increasing the molar mass is expected to decrease the amount of molecular crossover in RFB applications but, at the same time, to increase the viscosity of the final solution. In this section we investigate how variation of molar mass affects both parameters aiming to identify an optimal molar mass.

**Fig. 5:**
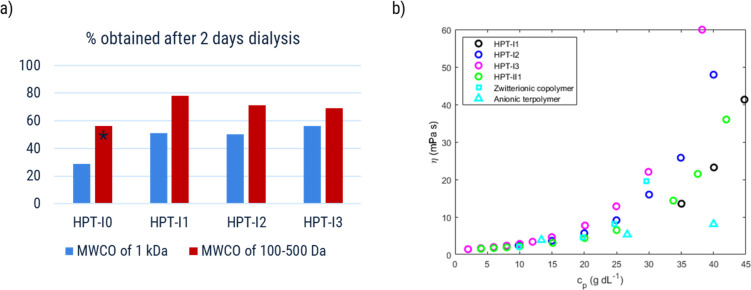
(a) Fraction of obtained material (% w/w) after dialysis for two days using membranes with a molecular weight cut-off of 1 000 Da (blue) and 100 to 500 Da (red). (b) Dynamic viscosity of the polymer solutions after ion exchange as a function of polymer concentration in 1.5 M aqueous NaCl solution. *This datapoint is affected by damage in one of the two dialysis tubes used for dialysis.

As was discussed in the synthesis section, in the last step an ion-exchange process was performed on the polymer samples using dialysis in a concentrated NaCl solution. After these processes, the samples corresponding to various molar masses are named as HPT-I*x*Cl with *x* = 0 to 3. The size exclusion of the polymer samples was evaluated by measuring the amount of obtained material after dialysis. Fig. 5a shows the results of the obtained materials for HPT-I0Cl to HPT-I3Cl using size-exclusion membranes with MWCOs of lower values (100 to 500 Da) and higher values (1 000 Da).

First, in all the cases, a significant amount of material (at least 22%) is lost after two days of dialysis. Considering the molar mass distribution from SEC and AUC (Fig. 1 and S13†), this fact can be understood based on the presence of a large fraction of low molar mass polymers (or oligomers) in all the samples. Results obtained from AUC reveal that even HPT-I3 with the highest average molar mass, has a significant amount of material with small sedimentation coefficients (Fig. S13†). The smaller species can pass the dialysis membrane, rationalizing the high amount of material loss in all cases. Using the membrane with the lower MWCO is shown to significantly improve the amount of obtained material (by 22% on average), indicating the presence of a significant fraction of polymers with sizes in the range of the pore size of the membrane with a MWCO of 1 000 Da. Regarding the effect of molar mass, by comparing HPT-I0 with HPT-I1, a significant increase in the amount of obtained polymer is observed with introduction of the trifunctional amine (29% and 56% for HPT-I0 compared to 51% and 78% for HPT-I1 using membranes with MWCO of 1 000 Da and 100 to 500 Da, respectively). However, with further addition of trifunctional amine in the synthesis (going from HPT-I1 to HPT-I3) the amount of obtained material shows insignificant changes.

Besides, dynamic viscosities of polymer solutions obtained after ion exchange and dialysis using the membrane with MWCO of 1 000 Da was measured in 1.5 M NaCl solutions to evaluate final applicability of the hyperbranched polymers in the redox flow battery electrolyte, as shown in Fig. 5b.

HPT-I2Cl and HPT-I3Cl were highly soluble in 1.5 M NaCl aqueous solutions enabling viscosity measurements in the entire range of concentrations. For HPT-I1Cl, however, small particles were found floating in the solution which were not dissolved with further dilution. While these particles did not impede the movement of the ball in the larger capillary of the viscometer, the data of which are shown in Fig. 5b, they interfered with the moving ball in case of the smaller capillary. The latter has a smaller gap between ball and capillary walls. In case of HPT-I0Cl (which was exceptionally obtained from dialysis with the membrane with MWCO of 100 to 500 Da) similar particles were observed with a higher content and, thus, HPT-I0Cl was considered insoluble and the corresponding viscosity data are not presented.

With increasing molar mass from HPT-I1Cl to HPT-I3Cl, the increase of viscosity with concentration becomes steeper, similar to the previously observed trend of viscosity for polymers in the neutral state. Overall, it can be concluded that using a trifunctional amine for chain extension has improved the solubility and suppressed crossover by comparing HPT-I1Cl and HPT-I0Cl. However, further increase in molar mass, from HPT-I1Cl to HPT-I3Cl, does not significantly impact crossover while causing a significant increase in solution viscosity. In fact, additional trifunctional amine molecules have a higher chance to react with large molecules possessing relatively high number of reactive groups. As a result of such reactivity, larger molecules grow even faster compared to the smaller molecules by addition of trifunctional amine causing a broader molar mass distribution with increased trifunctional amine content. Such behavior is observed in the molar mass distributions obtained by both SEC and sedimentation coefficient distribution from AUC measurements. As a result of such broad distributions, a small fraction of large macromolecules is formed which dominate the viscosity behavior through introduction of macromolecular entanglements. On the other hand, the major fraction of small molecules is not significantly affected by additional amounts of trifunctional amine. This fraction can transport through the membrane causing a high material loss *via* crossover.

Based on these results, HPT-I1Cl is chosen as the optimum point of viscosity and crossover behavior. A new sample (HPT-II1) was synthesized with a similar amount of trifunctional amine but terminated with 4-amino TEMPO instead of 1-methylpiperazine which resulted in an increased TEMPO content in each polymer chain compared to HPT-I1 (ESI S9†). For HPT-II1Cl, the ion exchange and dialysis were continued until no trace of iodide was observed in the sample (based on cyclic voltammetry). With extension of the dialysis time even more material was lost, which resulted in only about 10% of the obtained material. HPT-II1Cl revealed excellent solubility in 1.5 M aqueous NaCl. For comparison, examples of linear TEMPO-based polymers from literature are also plotted (cyan squares correspond to datapoints of a linear zwitterionic copolymer reported by Hagemann *et al.* and cyan triangles correspond to those of a linear anionic terpolymer reported by Fu and Zhang *et al.*).^7,14^ These data are calculated based on the concentrations provided in the respective publication (in A h L^−1^), the mass of the repeating unit for the provided structure, and the reported degree of oxidation. These two examples are selected among other polymer samples in the literature for multiple reasons. First, to our best knowledge, they represent the lowest reported viscosity values for TEMPO-based polymers which are soluble in aqueous solutions. Second, the selected supporting electrolyte solution (1.5 M NaCl) and the utilized method of viscosity measurement are similar to our case, enabling quantitative comparison. However, from the viewpoint of electrostatic interactions the selected examples are different. While HPT-II1Cl is a cationic polymer, the copolymer reported by Hagemann *et al.* is zwitterionic and the terpolymer reported by Fu and Zhang *et al.* is anionic. In the dilute region (1.2 ≤ *η*_r_ ≤ 2.5), the viscosity behavior of all new systems is comparable to the linear polymers reported earlier. With the increase of the concentration, differences in the viscosity become more prominent.

The terpolymer shows the lowest viscosities among the polymers revealing a viscosity of 8.1 mPas at a concentration of around 40 g dL^−1^. Next, the hyperbranched polymer HPT-II1Cl reveals a superior viscosity behavior compared to the zwitterionic copolymer. While the zwitterionic copolymer shows a viscosity of 20 mPas at a mass concentration of 29 g dL^−1^, HPT-II1Cl shows a lower viscosity (14 mPas) at a higher concentration of 34 g dL^−1^ and a slightly higher viscosity of 21 mPas at a mass concentration of 38 g dL^−1^. Furthermore, the molar mass of the repeating unit (one TEMPO monomer + one comonomer) in case of the linear zwitterionic copolymer is around 520 g mol^−1^ while in the case of HPT-II1Cl the calculated mass containing 1 mole of TEMPO is around 385 g mol^−1^ (for details of the calculations see ESI S10†) which is expected to further improve the applicable TEMPO concentration and corresponding electrolyte capacity compared to the previously reported linear polymer.

**Fig. 6:**
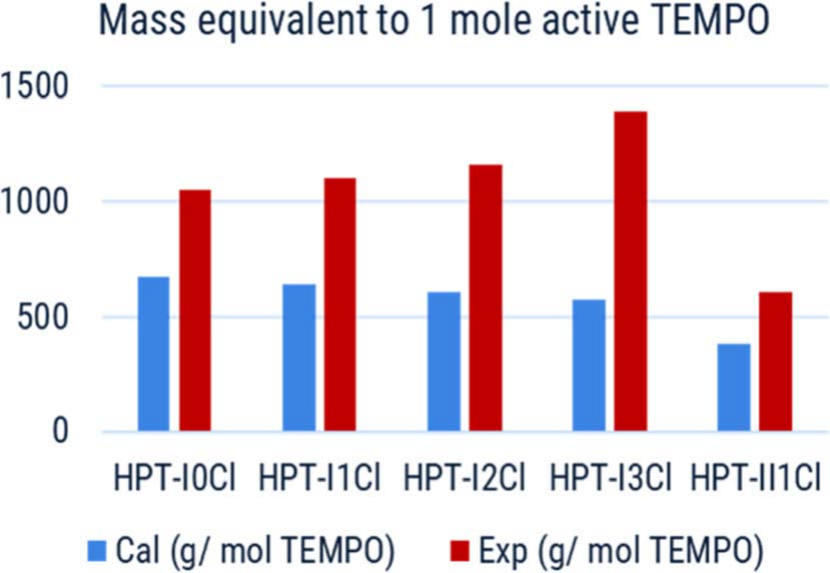
Calculated (blue) and EPR-based (red) mass of polymer samples equivalent to 1 mole active TEMPO.

However, the mass of the polymer samples equivalent to 1 mole TEMPO was also measured experimentally using EPR and compared with the calculated values, as shown in Fig. 6. In all cases the mass obtained from the EPR was higher than that of the calculated one based on the polymer repeating units by a factor of 1.6 to 2.4. One source of error can arise from the calculation of the incorporated AEP. In the calculations it is assumed that each AEP molecule reacts with three acrylamide groups but if the reactivity of the formed secondary amine is too low to consume the third acrylamide, the remaining double bond will be consumed in the termination step with 1-methylpiperazine or 4-amino TEMPO. In that case, the overall mass of polymer for each TEMPO unit would increase, in case of HPT-I1Cl to HPT-I3Cl, compared to the currently calculated value. However, we argue that this cannot be the only source of this difference because even in the sample without AEP, *i.e.*, HPT-I0Cl, where the repeating unit is exactly known, the experimental mass equivalent to one active TEMPO is higher than the mass of the repeating unit by a factor of 1.6. This can be explained by deactivation of the TEMPO moieties through a side reaction. To further investigate the cause of deactivation, the monomer (4-amino TEMPO) and the polymer HPT-II1 (before methylation) were also evaluated using EPR. The mass of 224 ± 32 g mol^−1^ corresponding to a ratio of EPR-based mass to the calculated mass of 1.31 was obtained for the monomer. The deviation from 1 can be explained by a combination of error margin in the EPR measurement and the presence of impurities in the monomer. For HPT-II1 before methylation a mass of 385 ± 19 g mol^−1^ corresponding to a ratio of 1.18 was obtained. This indicates that the major deactivation had not occurred during the polymerization as the TEMPO moieties are active after polymerization. One could, therefore, hypothesize that side reactions during the methylation reaction could potentially deactivate the TEMPO moieties. To this end, for future works, one direction of improvements can be to optimize the methylation reaction.

**Fig. 7:**
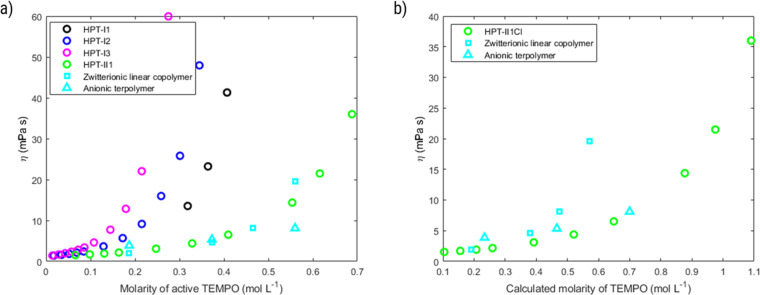
(a) Dynamic solution viscosity as a function of molarity of active TEMPO. Molarity of active TEMPO is based on the experimentally determined masses from Fig. 6. (b) Dynamic solution viscosity as a function of calculated molarity of TEMPO. Calculated molarity assumes that all the incorporated TEMPO units are active. For hyperbranched polymer, it corresponds to the calculated value of molar mass equivalent to 1 TEMPO in Fig. 6 and in case of the zwitterionic copolymer and the anionic terpolymer such value corresponds to an oxidation degree of 100%.

Based on the experimentally obtained equivalent masses, the viscosity of polymer solutions is also plotted as a function of active TEMPO molarity in Fig. 7a. In this case a superior behavior, *i.e.*, significantly higher accessible concentration range within the relevant viscosity window, of HPT-II1Cl is observed in comparison to HPT-I1Cl, HPT-I2Cl, and HPT-I3Cl. The highest concentration of HPT-II1Cl used herein is 0.69 M corresponding to a theoretical capacity of 18.5 A h L^−1^ which is superior compared to previously reported capacities of TEMPO-based polymer catholytes (ESI S16†). However, at a lower concentration, the viscosity value of HPT-II1Cl appears between those of the zwitterionic copolymer and the anionic terpolymer, *i.e.*, at a concentration of around 0.56 M active TEMPO (15 A h L^−1^), viscosities of 20 mPas, 14 mPas, and 8 mPas were observed for the zwitterionic copolymer, HPT-II1Cl, and the anionic terpolymer, respectively. Overall, it can be concluded that partial deactivation of TEMPO moieties limited the viscosity advantage of HPT-II1Cl by decreasing the concentration of active TEMPOs.

Fig. 7b displays an alternate view of such viscosity plots, in which it is assumed that all TEMPO moieties would be active. For HPT-II1Cl such case corresponds to the calculated molar masses equivalent to 1 mole TEMPO as in Fig. 6 (blue bar), while in cases of the zwitterionic copolymer and the anionic terpolymer it corresponds to an oxidation degree of 100%. By this view, we can reduce the discussion on pure effects of the viscosity behavior. In that case, HPT-II1Cl reveals similar properties to that of the anionic terpolymer, and a significantly improved behavior compared to the zwitterionic copolymer. At a calculated molarity of TEMPO of 0.57 mol L^−1^ the solution viscosity reached 20 mPas for the linear zwitterionic copolymer, while a solution viscosity of 21 mPas is obtained at a TEMPO molarity of 0.98 mol L^−1^ for the hyperbranched polymer system. In other words, if both polymer systems would contain 100% activity of TEMPO, the branched system allows for approximately 70% more active TEMPO at a dynamic viscosity value of around 20 mPas (Fig. 7b).

### Electrochemical properties: cyclic voltammetry and step potential chronoamperometry

In this section the electrochemical properties of the hyperbranched TEMPO polymers are discussed. The selected candidate to evaluate the electrochemical activity is HPT-II1Cl because of the higher content of incorporated TEMPO in the molecules, but the results of HPT-I2Cl and HPT-I3Cl are also available in ESI S11† for comparison.

**Fig. 8:**
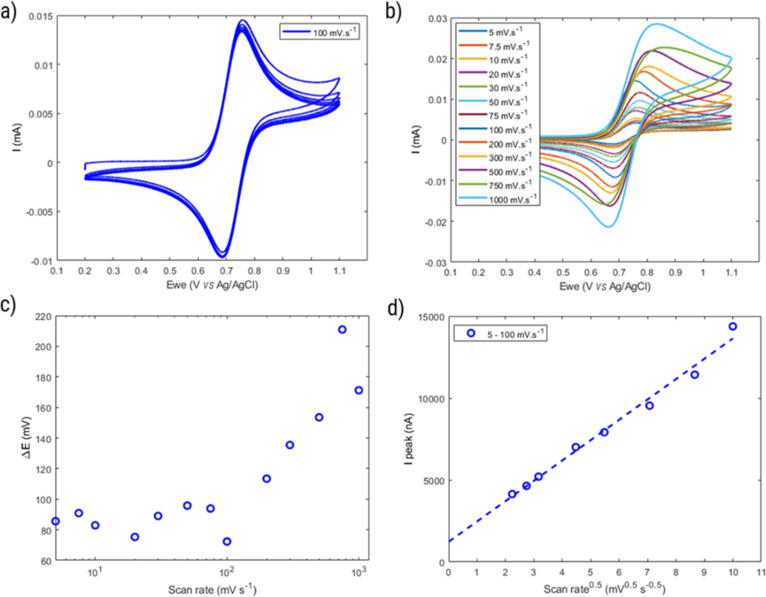
(a) Cyclic voltammogram of HPT-II1Cl (5.7 mM active TEMPO) in 1.5 M NaCl aqueous solution at pH = 2.0 and at the scan rate of 100 mV s^−1^ (six consecutive cycles). (b) First cycle voltammograms at various scan rates. (c) Peak-to-peak potential difference (obtained from the first cycle voltammograms) as a function of scan rate. (d) Oxidation peak current as a function of square root of scan rate in the reversible range (scan rates of 5 to 100 mV. s^−1^).

Fig. 8 displays the behavior of HPT-II1Cl in cyclic voltammetry at a scan rate of 100 mV s^−1^ for six consecutive cycles. Here, to prevent any interference of the remaining tertiary amine groups in the electrochemical activity, we have conducted cyclic voltammetry at pH = 2.0 to ensure that any remaining tertiary amine groups of the polymer are protonated and electrochemically inactive. To prevent the influence of migration on the electrochemical behavior of HPT-II1Cl, an electrolyte concentration of 1.5 M NaCl was used similar to the targeted solution for viscosity and battery experiments. Furthermore, a standard potential of *E*^0^ = 0.72 V *vs.* Ag/AgCl was obtained, which is within the range of other TEMPO derivatives (examples are provided in the ESI S14†). The redox potential of a redox-active moiety can be shifted by the effect of electron-donating or electron-withdrawing groups. In most previously reported TEMPO polymers for redox flow batteries, a methacrylate-based monomer was used for the polymerization in which the TEMPO moiety is attached to the backbone *via* an ester group.^7,8,14^ Two of them reported an *E*^0^ value of 0.7 *vs.* Ag/AgCl, which is slightly lower compared to our case.^7,8^ This is attributed to the difference in electron-withdrawing effect of the group on the 4-position of the TEMPO ring (quaternary ammonium in HPT-II1Cl compared to the ester group in the methacrylate-based polymers). *N*,*N*,*N*-2,2,6,6-Heptamethylpiperidinyloxy-4-ammonium chloride (TMATEMPO) small molecule with a quaternary ammonium reveals a higher redox potential of *E*^0^ = 0.79 V *vs.* Ag/AgCl.^45^ A peak potential separation (Δ*E*) of 72 mV and an anodic to cathodic peak current ratio of 
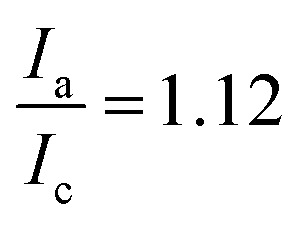
 represents an electrochemically quasi-reversible behavior at this scan rate.

Moreover, the properties of HPT-II1Cl are evaluated as a function of cycling scan rate from 5 mV s^−1^ to 1 000 mV s^−1^, as shown in Fig. 8b. Δ*E* is plotted as a function of scan rates in Fig. 8c. Here, Δ*E* fluctuates around a value of 80 mV at scan rates up to 100 mV s^−1^. However, with further increase Δ*E* increases reaching up to around 200 mV at a scan rate of 750 mV s^−1^. In Fig. 8d, the peak oxidation current is plotted as a function of square root of the scan rate. If electrochemical reversibility is assumed, eqn (2) can be applied to this relation from which a diffusion coefficient of *D* = 1.6 × 10^−6^ cm^2^ s^−1^ is estimated. This value is three times lower than the diffusion coefficient reported for the TMATEMPO small molecule (4.8 × 10^−6^ cm^2^ s^−1^), but higher than the previously reported values for other TEMPO-based polymers (see the ESI S15† for comparison).

This faster diffusion arises from the same origin as the discussed viscosity advantage, namely the more compact structure of hyperbranched polymers and the lower molar mass compared to the previously reported linear polymer. Herein, it should be noted that the diffusion coefficient obtained based on the electrochemical methods are derived based on the theories which are mainly developed for molecules with one redox-active moiety. In the case of polymers, each molecule has multiple redox-active moieties and, thus, when the molecule is transported through diffusion to the electrode surface, there are multiple redox-active moieties that can react at the electrode surface. Therefore, the diffusion coefficient obtained here is not necessarily the diffusion coefficient of polymer molecules but the result of polymer chain diffusion and multiple reactions at the surface. In that sense the reported value is an apparent diffusion coefficient reflecting the transport of each redox-active moiety.

**Fig. 9:**
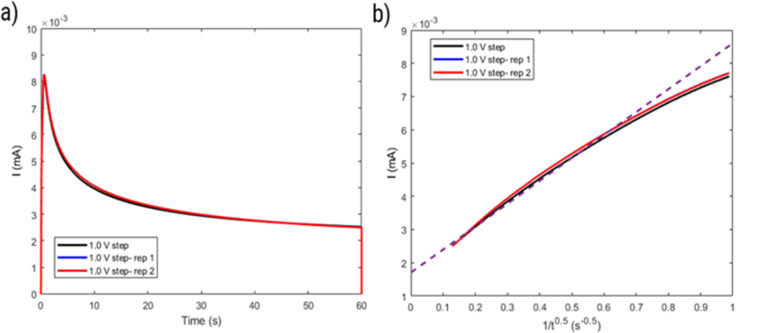
(a) Current as a function of time and (b) current as a function of inverse square root of time obtained from a step potential chronoamperometry test on HPT-II1Cl (5.7 mM active TEMPO) in 1.5 M NaCl solution at pH = 2.0.

However, considering the higher Δ*E* of the polymer (around 80 mV) compared to a reversible process (around 59 mV), the behavior of HPT-II1Cl may deviate from eqn (2). Thus, the diffusion coefficient obtained herein is later compared to those values based on other electrochemical experiments (CA and RDE), which do not rely on electrochemical reversibility. Fig. 9 displays the result of potential step chronoamperometry for HPT-II1Cl at the similar concentration of 5.7 mM in 1.5 M NaCl solution at pH = 2.0, where different colors show the results of the repetitions of the same experiment. Application of a step potential of 1.0 V starts the oxidation of the TEMPO moieties in the polymers at the electrode surface, which is described by the Cottrell equation (eqn (3)). The measured current at the electrode surface decreases over time due to an increasing thickness of the diffusion layer. In case of semi-infinite diffusion, the current scales inversely with the square root of time. Under most experimental conditions, natural convection limits the thickness of the diffusion layer and, thus, a deviation from the semi-infinite diffusion is observed for long step durations. Anyhow, as observed in Fig. 9b, a nearly linear behavior is observed in the time range from 1 to 60 seconds. Therefore, a linear regression is built for this range and from its slope, the diffusion coefficient is estimated as *D* = 1.2 × 10^−6^ cm^2^ s^−1^, which is close to the value obtained from cyclic voltammetry.

### Electrochemical properties: rotating disc electrode voltammetry

**Fig. 10:**
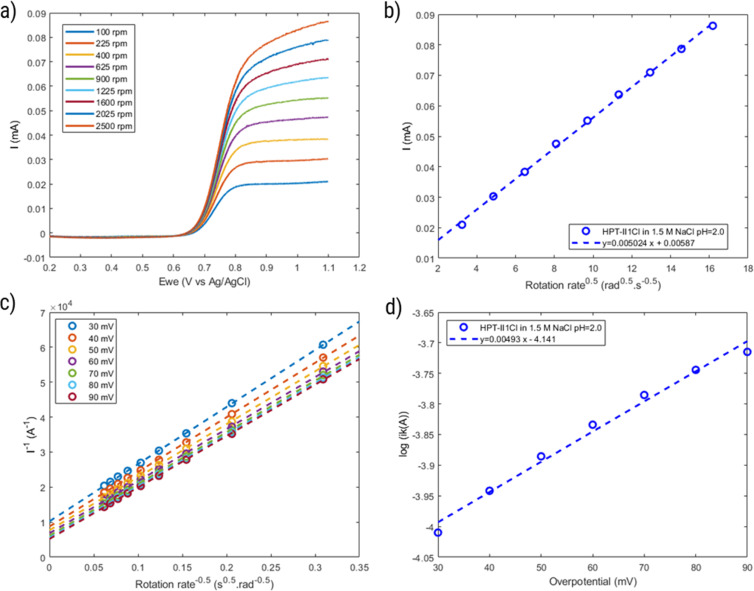
(a) Voltammograms of HPT-II1Cl (2.0 mM) in 1.5 M NaCl solution at pH = 2.0 using rotating disc electrode at various rotation speeds (the background current is subtracted, using separately measured voltammograms in 1.5 M NaCl solution). (b) Limiting currents (measured at 1.1 V) as a function of square root of the rotation speed. (c) Inverse current as a function of inverse square root of the rotation speed at various overpotentials (potential differences from the formal potential). (d) Logarithm of mass-transfer-independent kinetic current as a function of the potential difference from the formal potential.

To further investigate the electrochemical properties of HPT-II1Cl, rotating disc electrode voltammetry is conducted, as shown in Fig. 10. The voltammograms of the background solution (1.5 M NaCl) at similar rotation rates were subtracted from the results of the polymer solution. With increasing rotation rate, the measured current at the electrode surface increases due to decreasing the thickness of the diffusion layer. The limiting currents measured at 1.1 V reveals a linear dependency on the square root of the rotation rate as described by Levich equation (eqn (4)). From the slope of this curve, a diffusion coefficient of *D* = 1.0 × 10^−6^ cm^2^ s^−1^ is obtained. This value of diffusion coefficient is close to the values obtained using CV (*D* = 1.6 × 10^−6^ cm^2^ s^−1^) and CA (*D* = 1.2 × 10^−6^ cm^2^ s^−1^).

Comparing diffusion coefficients obtained from CV and RDE, it is worth mentioning that application of eqn (2) to CV data requires electrochemical reversibility in the investigated scan rates, while with RDE such conditions are not required. Furthermore, in our experiments, a blank subtraction was performed for RDE data evaluation, while for CV, the current of the blank solution was not subtracted, which might slightly influence the peak currents due to the presence of capacitive currents.

Furthermore, the inverse current is plotted as a function of inverse square root of rotation rates, which shows a linear relationship, following the Koutecký–Levich equation (eqn (5)), as shown in Fig. 10c. From the intercept of this linear relation, the mass-transfer-independent kinetic currents (*I*_k_) can be obtained at various potentials with respect to standard potential. Finally, from the logarithmic plot of *I*_k_*versus* the potential difference from the standard potential, as shown in Fig. 10d, the electrochemical reaction rate constant (*k*^0^ = 1.9 × 10^−3^ cm s^−1^) and the transfer coefficient (*α* = 0.71) are obtained. This value of the electrochemical rate constant is lower than that of the small molecule TMATEMPO (*k*^0^ = 4.2 × 10^−3^ cm s^−1^) but higher than those of the previously reported TEMPO polymers (see ESI S15†) including the zwitterionic copolymer (9.7 × 10^−4^ cm s^−1^) and the anionic terpolymer (7.8 × 10^−4^ cm s^−1^).^7,14,45^ The fact that polymers reveal a lower heterogenous charge transfer rate than that of small molecules has been attributed to their limited Brownian motion.^46^ In addition, it was reported that long and flexible alkyl chains could improve the charge transfer rate. Based on that, the higher charge transfer rate of HPT-II1Cl compared to methacrylate-based linear polymers can similarly be attributed to the higher mobility of the TEMPO moieties in HPT-II1Cl, which are attached to the backbone *via* three sp^3^ atoms before the carbonyl group, compared to methacrylate-based polymers where TEMPO is directly attached to the ester group (1 sp^3^ atom between the TEMPO moiety and the carbonyl group).

Overall, using cyclic voltammetry, chronoamperometry, and rotating disc electrode voltammetry, a quasi-reversible oxidation/reduction for the TEMPO-containing hyperbranched polymer with a higher diffusion coefficient and a faster heterogenous charge transfer rate compared to previously reported TEMPO-based linear polymers is demonstrated.

### Application in redox flow battery

**Fig. 11:**
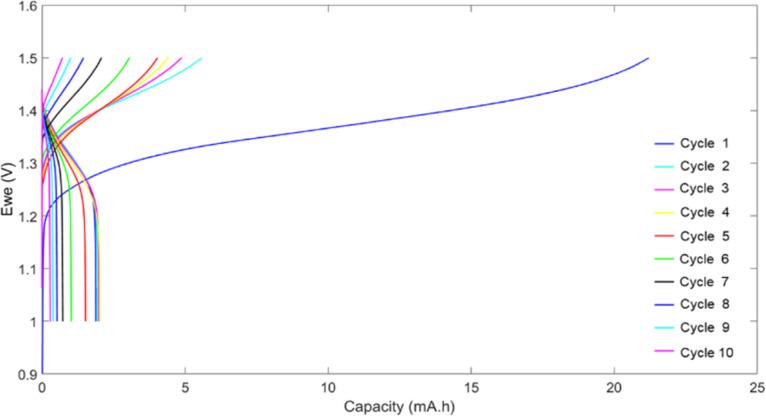
Cell voltage and capacity during the first 10 cycles of galvanostatic charging and discharging using 10 mL solution of HPT-II1Cl (18.65 mM active TEMPO in 1.5 M NaCl solution at pH = 2.0 corresponding to theoretical capacity of 5 mAh) as the catholyte and 25 mL solution of MV (18.65 mM in 1.5 M NaCl solution at pH = 2.0) as the anolyte at the current of 5 mA.

To evaluate the behavior of the hyperbranched polymer in redox flow batteries, HPT-II1Cl is applied as the catholyte against MV as the anolyte, both dissolved in 1.5 M NaCl at pH = 2.0. The battery was charged and discharged galvanostatically at a constant current of 5 mA with low and high voltage cut-off limits of 1.0 V and 1.5 V, respectively. Fig. 11 displays the voltage and the capacity during charging and discharging for consecutive cycles. In the first cycle, charging continued up to around 21 mAh, which was significantly beyond the calculated theoretical capacity of 5.0 mAh (based on the active TEMPO moieties). This charging capacity was irreversible as only a capacity of around 2.0 mAh was obtained during discharging. TEMPO moieties are normally expected to show reversible oxidation and reduction with acceptable stability over a few days.^47^ Therefore, such abnormal behavior of the first cycle could indicate a chemical side reaction interfering with the redox activity of the TEMPO moieties. From this point, the behavior of the battery during the next cycles was difficult to explain partly because of the presence of unknown products of the overoxidation of the catholyte. For the second cycle, the charging capacity was decreased to 5.6 mAh, which is around the expected theoretical capacity, and discharging still occurs only to a capacity of about 2 mAh. In the next cycles, a fast-decaying capacity is observed resulting in less than 20% of the initial discharge capacity in the 9th cycle. To explain the overoxidation of the catholyte side, we further investigated three possible causes.

The first scenario is the overreduction of the TEMPO moieties through side reactions in the methylation reaction. As was discussed based on EPR results, the TEMPO moieties were partially deactivated, and the deactivation could be due to overreduction through reaction with iodide in the methylation reaction. To mitigate this issue, a new batch of polymer, similar to HPT-II1, was synthesized. This time chloromethane was utilized for the methylation reaction instead of iodomethane. The resulting methylated polymer was characterized using EPR from which a mass equivalent to 1 mole TEMPO of 557 ± 33 g mol^−1^ was obtained, which is slightly lower compared to HPT-II1Cl. A battery test under similar conditions was performed utilizing this polymer (results are presented in the ESI, S13†). However, even this polymer revealed a similar behavior with an overcharging in the first cycle followed by a low discharge capacity and a fast capacity decay like the HPT-II1Cl material. Therefore, one can conclude that possible side reactions of iodide during the methylation reaction were not the main cause of the overoxidation of the TEMPO moieties under battery conditions.

The second possible cause of the overcharging could be oxidation of the remaining tertiary amines in the polymer chain after methylation. Although the methylation reaction was quite efficient (as described in ESI S7† for HPT-I0 to HPT-I3), a fraction of tertiary amines is expected to remain. Those can undergo irreversible oxidation. As discussed in the ESI S6,† the deprotonated tertiary amines of the polymer start to get oxidized at potentials higher than 0.8 V *vs.* Ag/AgCl, while the protonation at low pH can prevent such side reactions. Although the battery tests were started at pH = 2.0 to prevent such oxidation, the pH value of the system could increase during cycling if hydrogen evolution reaction had consumed the hydronium ions on the anolyte side. Sampling of the catholyte after cycling revealed the pH value still being 2.0 and, thus, the possibility of deprotonation and oxidation of the tertiary amines can be ruled out.

The third possibility is the interaction of the polymer backbone with the oxidized TEMPO. Previous reports of small molecules and polymers containing TEMPO and amide moieties have shown reasonable stability and, thus, the amide groups of the polymer backbone are not expected to interfere with the redox activity of the TEMPO moieties.^48,49^ Therefore, it is expected that other chemical interactions with, for example, protonated amine groups, triazine rings, *etc.* are responsible for such TEMPO degradation. Understanding the underlying mechanism requires further investigations.

Another possible parasitic reaction can be the disproportionation of TEMPO moieties in acidic medium.^50^ However, comparing the voltammogram of the TEMPO polymer kept at a pH = 2.0 without cycling to a similar solution after cycling shows that cycling was the main cause of the TEMPO degradation but not keeping the TEMPO polymer at pH = 2.0 (ESI S13†).

Overall, the battery performance of the hyperbranched TEMPO polymer indicates the presence of a side reaction which degrades the TEMPO moieties during cycling. Here we analyzed three possible scenarios, from which we excluded two: (i) overreduction of the TEMPO moieties by iodide during methylation and (ii) interference of the oxidation of the remaining tertiary amine groups. However, to understand the mechanism of such degradation, designing symmetrical battery experiments with further chemical characterizations are suggested which is out of the scope of this work.

## Conclusions

In this work, hyperbranched redox-active polymers were introduced as potential low-viscosity electrolytes for redox flow battery applications. A step-growth polymerization method based on an aza-Michael addition using 4-amino TEMPO and a triacrylamide was reported, followed by a post-polymerization modification and ion exchange to further improve the solubility in aqueous solutions. Through investigation of the Kuhn–Mark–Houwink–Sakurada (KMHS) relation, the compact structure of the synthesized hyperbranched polymers was confirmed. The hydrodynamic behavior of the polymers was studied *via* viscometry as a function of molar mass and concentration. It was found that chain extension *via* addition of a trifunctional amine increases the molar mass and viscosity of the solutions, especially at high concentrations.

Additionally, potential applicability of the polymers with size-exclusion membranes was evaluated by measuring the amount of material crossover in *ex situ* dialysis experiments using membranes with various MWCOs. Overall, a high amount of material loss was observed (between around 20 to 70% w/w depending on the sample molar mass and the membrane MWCO) after only two days of dialysis followed by an even higher loss when dialysis was continued longer. Therefore, material crossover remained as one of the main challenges of the hyperbranched polymers, which is mainly attributed to the broad distribution of the molar mass in the synthesized polymers. Interestingly, a significant decrease in crossover was obtained through introduction of the trifunctional amine in polymerizations. Based on the results of crossover and viscosity, an optimum amount of added trifunctional amine was found and a hyperbranched polymer with the selected amount of trifunctional amine and additionally incorporated TEMPO moieties was synthesized.

Although the selected polymer possessed an overall high concentration of TEMPO with relatively moderate viscosities (around 1 M TEMPO, according to the calculated mass of the repeating unit, at a viscosity of 21 mPas), possible partial deactivation of TEMPO moieties decreased the concentration of active TEMPO moieties leading to a viscosity behavior in the range of previously reported linear polymers.

The electrochemical properties of the selected polymer were investigated *via* cyclic voltammetry, chronoamperometry, and rotating disc electrode voltammetry. An electrochemically quasi-reversible redox activity with faster diffusion and higher heterogenous charge transfer rate compared to previously reported linear polymers was observed. In the galvanostatic battery cycling test, however, the resulting polymer revealed a poor performance with an irreversible charging in the first cycle, followed by a fast-decaying capacity and low coulombic efficiencies, which are mainly attributed to the side reactions of the oxidized TEMPO moieties.

To sum up, a new synthesis and thorough hydrodynamic and electrochemical characterization of hyperbranched TEMPO-based polymers were presented, which enabled decreased viscosity and faster diffusion and charge transfer rate compared to linear polymers. Nevertheless, the main challenges of being prone to crossover, partial deactivation of the TEMPO moieties, and subsequent side reactions represent remaining challenges with regard to their application in redox flow batteries.

In future studies, these challenges can be addressed through various strategies. To mitigate the crossover problem, one idea can be to choose multifunctional amines (*e.g.*, diethylenetriamine) instead of trifunctional amine to further increase the molar mass at the same molar ratio. Alternatively, multifunctional alkyl halides (*e.g.*, 1,3-dichloropropane) can be used to further increase molar mass during the methylation reaction by linking the polymer molecules together on their amine groups. Deactivation of TEMPO molecules should also be further investigated. Alternative methylation procedures using, *e.g.*, dimethyl carbonate can be considered to further optimize the methylation reaction.

## Data availability

The data supporting this contribution are available from the corresponding author upon reasonable request.

## Author contributions

Koosha Ehtiati: conceptualization (lead), investigation (lead), data curation (lead), formal analysis (lead), validation (lead), visualization (lead), writing – original draft (lead). Ilya Anufriev: conceptualization (supporting), investigation (supporting), writing – original draft (supporting), writing – review and editing. Christian Friebe: conceptualization (supporting), investigation (supporting), writing – review and editing. Ivan A. Volodin: conceptualization (supporting), investigation (supporting), writing – review and editing. Christian Stolze: conceptualization (supporting), writing – review and editing. Simon Muench: investigation (supporting), writing – review and editing. Grit Festag: conceptualization (supporting), investigation (supporting), writing – review and editing. Ivo Nischang: conceptualization (supporting), supervision, resources, writing – original draft (supporting), writing – review and editing. Martin D. Hager: conceptualization (supporting), supervision, funding acquisition, resources, writing – review and editing. Ulrich S. Schubert: conceptualization (supporting), supervision, funding acquisition, writing – review and editing.

## Conflicts of interest

There are no conflicts to declare.

## Supplementary Material

RA-014-D4RA03925D-s001

## References

[cit1] Albertus P., Manser J. S., Litzelman S. (2020). Joule.

[cit2] Viswanathan V., Mongird K., Franks R., Li X., Sprenkle V., Baxter R. (2022). Energy.

[cit3] Minke C., Turek T. (2018). J. Power Sources.

[cit4] Amini K., Shocron A. N., Suss M. E., Aziz M. J. (2023). ACS Energy Lett..

[cit5] Gao M., Salla M., Song Y., Wang Q. (2022). Angew. Chem..

[cit6] Jiang H., Sun J., Wei L., Wu M., Shyy W., Zhao T. (2020). Energy Storage Mater..

[cit7] Hagemann T., Strumpf M., Schröter E., Stolze C., Grube M., Nischang I., Hager M. D., Schubert U. S. (2019). Chem. Mater..

[cit8] Janoschka T., Martin N., Martin U., Friebe C., Morgenstern S., Hiller H., Hager M. D., Schubert U. S. (2015). Nature.

[cit9] Lai Y. Y., Li X., Zhu Y. (2020). ACS Appl. Polym. Mater..

[cit10] Chai J., Wang X., Lashgari A., Williams C. K., Jiang J. (2020). ChemSusChem.

[cit11] Kozhunova E. Y., Gvozdik N. A., Motyakin M. V., Vyshivannaya O. V., Stevenson K. J., Itkis D. M., Chertovich A. V. (2020). J. Phys. Chem. Lett..

[cit12] Winsberg J., Muench S., Hagemann T., Morgenstern S., Janoschka T., Billing M., Schacher F. H., Hauffman G., Gohy J.-F., Hoeppener S. (2016). Polym. Chem..

[cit13] Yan W., Wang C., Tian J., Zhu G., Ma L., Wang Y., Chen R., Hu Y., Wang L., Chen T. (2019). Nat. Commun..

[cit14] Fu H., Zhang C., Wang H., Du B., Nie J., Xu J., Chen L. (2022). J. Power Sources.

[cit15] Winsberg J., Janoschka T., Morgenstern S., Hagemann T., Muench S., Hauffman G., Gohy J. F., Hager M. D., Schubert U. S. (2016). Adv. Mater..

[cit16] Boris D. C., Colby R. H. (1998). Macromolecules.

[cit17] Dobrynin A. V., Colby R. H., Rubinstein M. (1995). Macromolecules.

[cit18] Rubinstein M., Colby R. H., Dobrynin A. V. (1994). Phys. Rev. Lett..

[cit19] Chen H., Kong J. (2016). Polym. Chem..

[cit20] Jikei M., Kakimoto M.-a. (2001). Prog. Polym. Sci..

[cit21] Perevyazko I., Seiwert J., Schömer M., Frey H., Schubert U. S., Pavlov G. M. (2015). Macromolecules.

[cit22] Yates C., Hayes W. (2004). Eur. Polym. J..

[cit23] McKee M. G., Unal S., Wilkes G. L., Long T. E. (2005). Prog. Polym. Sci..

[cit24] McKee M. G., Wilkes G. L., Colby R. H., Long T. E. (2004). Macromolecules.

[cit25] Sendijarevic I., Liberatore M. W., McHugh A. J., Markoski L. J., Moore J. S. (2001). J. Rheol..

[cit26] Schöniger W. (1956). Microchim. Acta.

[cit27] Nischang I., Perevyazko I., Majdanski T., Vitz J. r., Festag G., Schubert U. S. (2017). Anal. Chem..

[cit28] Kratky O. , Leopold H. and Stabinger H., in Methods in enzymology, Elsevier, 1973, vol. 27, pp. 98–110 10.1016/s0076-6879(73)27007-6 4797943

[cit29] Schuck P. (2000). Biophys. J..

[cit30] Compton R. G. and Banks C. E., Understanding Voltammetry, World Scientific, 2018

[cit31] Bard A. J., Faulkner L. R. (2001). Electrochem. Methods.

[cit32] Gao C., Yan D. (2004). Prog. Polym. Sci..

[cit33] Gao C., Yan D. (2001). Chem. Commun..

[cit34] van Benthem R. A., Meijerink N., Gelade E., de Koster C. G., Muscat D., Froehling P. E., Hendriks P. H., Vermeulen C. J., Zwartkruis T. J. (2001). Macromolecules.

[cit35] Yan D., Gao C. (2000). Macromolecules.

[cit36] Wang D., Liu Y., Hong C. Y., Pan C. Y. (2005). J. Polym. Sci., Part A: Polym. Chem..

[cit37] Wu D., Liu Y., He C., Chung T., Goh S. (2004). Macromolecules.

[cit38] Lezov A., Gubarev A., Kaiser T., Tobaschus W., Tsvetkov N., Nischang I., Schubert U. S., Frey H., Perevyazko I. (2020). Macromolecules.

[cit39] Hao J., Jikei M., Kakimoto M.-a. (2003). Macromolecules.

[cit40] Kong J., Schmalz T., Motz G. n., Müller A. H. (2011). Macromolecules.

[cit41] Tackx P., Tacx J. (1998). Polymer.

[cit42] Voit B. I., Lederer A. (2009). Chem. Rev..

[cit43] Grube M., Leiske M. N., Schubert U. S., Nischang I. (2018). Macromolecules.

[cit44] Grube M., Perevyazko I., Heinze T., Schubert U. S., Nischang I. (2020). Carbohydr. Polym..

[cit45] Janoschka T., Martin N., Hager M. D., Schubert U. S. (2016). Angew. Chem., Int. Ed..

[cit46] Sato K., Ichinoi R., Mizukami R., Serikawa T., Sasaki Y., Lutkenhaus J., Nishide H., Oyaizu K. (2018). J. Am. Chem. Soc..

[cit47] Rohland P., Nolte O., Schreyer K., Görls H., Hager M. D., Schubert U. S. (2022). Mater. Adv..

[cit48] Schröter E., Stolze C., Meyer J., Hager M. D., Schubert U. S. (2023). ChemSusChem.

[cit49] Schröter E., Stolze C., Saal A., Schreyer K., Hager M. D., Schubert U. S. (2022). ACS Appl. Mater. Interfaces.

[cit50] Ma Y., Loyns C., Price P., Chechik V. (2011). Org. Biomol. Chem..

