# Supplementary material for: Hyperbranched TEMPO-based polymers as catholytes for redox flow battery applications
Source: RSC Adv. 2024 Oct 18;14(45):32893–910. doi: 10.1039/d4ra03925d (PMC11487510; doi:10.1039/d4ra03925d)
Supplement: RA-014-D4RA03925D-s001 [file RA-014-D4RA03925D-s001.pdf]

Supplementary Information (SI) for RSC Advances.
This journal is © The Royal Society of Chemistry 2024

Supporting Information

Hyperbranched TEMPO-based Polymers as Catholytes for Redox Flow Battery Applications

Koosha Ehtiati,[a,b] Ilya Anufriev,[a,c] Christian Friebe,[d] Ivan A. Volodin,[a,b] Christian Stolze,[a,b] Simon Muench,[a,b] Grit Festag,[a,c] Ivo Nischang, [a,b,d,e] Martin D. Hager,[a,b,c,d] and Ulrich S. Schubert*[a,b,c,d]

Contents

S1: Kinetics of aza-Michael addition of cyclohexylamine and trimethylolpropane triacrylate 2

S2: Transesterification and low molar mass resulting from acrylate groups..3

S3: Kinetics of aza-Michael addition of cyclohexylamine and triacrylamide 5

S4: Molar mass investigation of the model system (polymer of cyclohexylamine and triacrylamide) 6

S5: Structural characterization of hyperbranched TEMPO-based polymers 7

S6: Electrochemical interference of tertiary amines and I^-..9

S7: Verification of the methylation reaction 12

S8: Hydrodynamic characterization using analytical ultracentrifugation (AUC)..14

S9: Synthesis route for preparation of HPT-II1Cl 17

S10: Calculation of the polymer mass equivalent to 1 mole active TEMPO 18

S11: Electrochemical properties of HPT-I2Cl and HPT-I3Cl...19

S12: Angular dependence of the viscosity data...22

S13: Further analysis of the battery performance..23

S14: Redox potential of other TEMPO derivatives for comparison 26

S15: Diffusion coefficient and charge transfer rate constant of TEMPO-based polymers for comparison28

S16: Theoretical and actual capacity of TEMPO-based polymer catholytes for comparison 29

References 30

[a] Laboratory of Organic and Macromolecular Chemistry (IOMC), Friedrich Schiller University Jena, Humboldtstrasse 10, Jena 07743, Germany.
[b] Center for Energy and Environmental Chemistry Jena (CEEC Jena), Friedrich Schiller University Jena, Philosophenweg 7a, Jena 07743, Germany.
[c] Jena Center for Soft Matter (JCSM), Friedrich Schiller University Jena, Philosophenweg 7, Jena 07743, Germany.
[d] Helmholtz Institute for Polymers in Energy Applications Jena (HIPOLE Jena), Lessingstr. 12 – 14, 07743 Jena, Germany.
[e] Helmholtz-Zentrum Berlin für Materialien und Energie GmbH (HZB), Hahn-Meitner-Platz 1, 14109 Berlin, Germany.

S1: Kinetics of aza-Michael addition of cyclohexylamine and trimethylolpropane triacrylate

To study the kinetics of an aza-Michael addition and its potential for polymerization, we started polymerization of a model system using cyclohexylamine and trimethylolpropane triacrylate (from now on is referred to as triacrylate) in 1:1 ratio in methanol as solvent at room temperature (RT). **Figure S1** shows the ^{1}H NMR-based conversion of NH groups and that of the double bonds in aza-Michael addition of the triacrylate and cyclohexylamine.

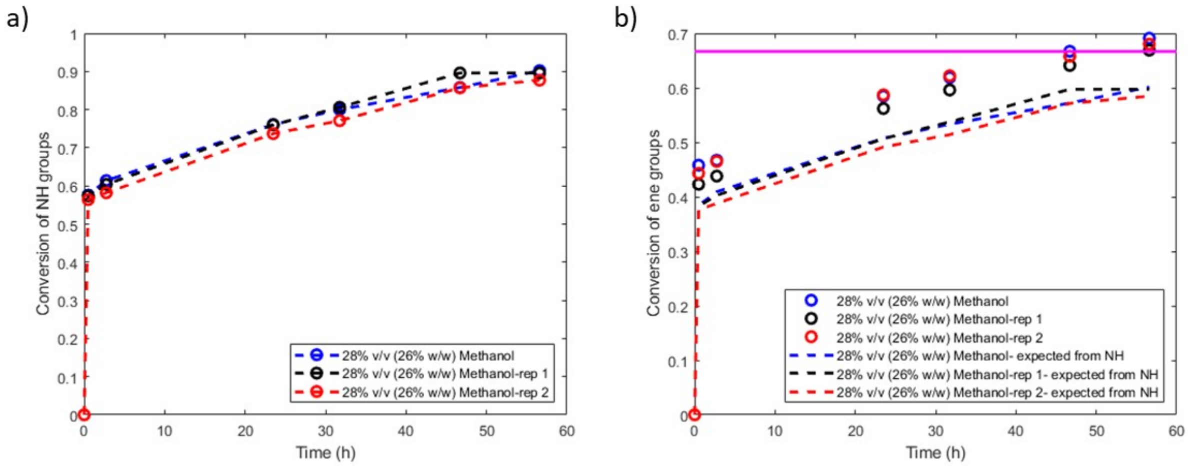

Figure S 1. *a) Conversion of the NH groups from cyclohexylamine and b) conversion of the double bonds from trimethylolpropane triacrylate as a function of time in polymerization of trimethylolpropane triacrylate and cyclohexylamine using aza-Michael addition. Various colors correspond to various batches of the same polymerization and the dashed lines in* ***Figure S1b*** *are the calculated values of the conversion of the double bonds solely based on the conversion of NH groups and the magenta line shows the maximum calculated conversion of ene groups based on that of NH groups if all the NH groups were reacted.*

Figure S1a shows that around half of the NH groups react relatively fast while the reaction of the remaining NH groups continues over the course of a few days. This is expected based on the higher reactivity of primary amines compared to formed secondary amines in the aza-Michael addition.[1] **Figure S1b** displays the corresponding conversion of the double bonds. Since the reactants were added in a 1:1 molar ratio, the conversion of the two functional groups should be related as $X_{ene} = \frac{2}{3}X_{NH}$, if the aza-Michael addition is the only reaction occurring in the system. Conversion of the double bonds follow the trend of the conversion of the NH groups but the experimentally obtained conversion of the double bonds are slightly higher than those of the calculated ones based on the conversion of the NH groups, indicating that a minor side reaction consumes the additional amount of double bonds. Anyhow, for a step-growth polymerization, high conversions are required which could be obtained by running the reaction for a few days. However, due to the low molar mass of the resulting polymers (discussed in details in section S2), triacrylates were not selected as the reactant but instead a triacrylamide (1,3,5-triacryloylhexahydro-1,3,5-triazine, from now on it is referred to as triacrylamide) was considered.

S2: Transesterification and low molar mass resulting from acrylate groups

Following the procedure described in Section S1, a polymer was synthesized after four days of polymerization with a final conversion of around 97% corresponding to a degree of polymerization around 16 and an average molar mass (M_n) of around 8,000 g mol^{-1}. The polymerization was terminated using an excess amount of 1-methylpiperazine. However the molar mass distribution, measured by SEC, showed a major fraction with low molar mass, overlapping with the signal of the eluent (around 31 mL), and a significantly decreased molar mass after methylation as shown in **Figure S2**.

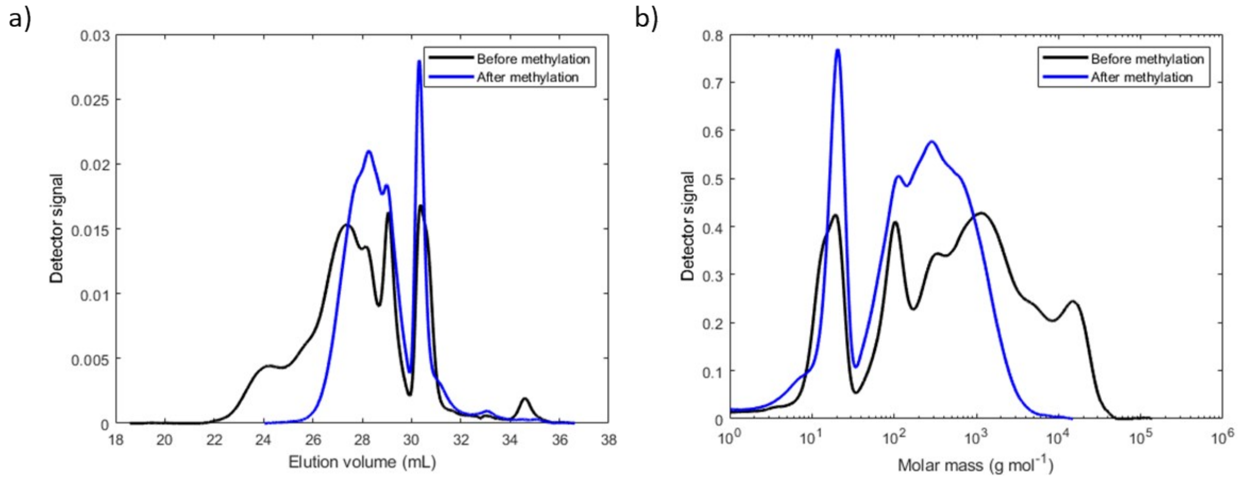

Figure S 2. a) Elugram and b) molar mass distribution of a polymer resulted from polymerization of cyclohexylamine and trimethylolpropane triacrylate before (black) and after (blue) methylation. SEC was conducted in an aqueous solution (0.1 M NaCl, 0.3 % trifluoroacetic acid) with poly(2-vinylpyridine) as the standard.

Figure S2a displays the elugram of the polymer before and after methylation reaction. The polymer peak is located near the peak of the eluent (at around 31 mL). The molar mass distribution also shows a peak around 1,000 g mol^{-1} (before methylation) and around 300 to 400 g mol^{-1} after methylation. This means that although the conversion was high, the obtained molar mass was low indicating the presence of a side reaction that cleaved the chains to lower molar mass molecules. We hypothesized that transesterification of the ester groups, along the backbone of the polymer, with methanol (the polymerization solvent) is the cause of such cleavage, as shown in **Scheme S1**.

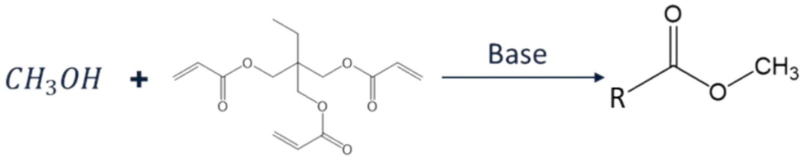

***Scheme S 1**. Schematic representation of the transesterification of the esters in the triacrylate monomer or the polymer backbone with methanol under basic conditions.*

To verify this hypothesis, ^{1}H NMR data of the polymerization batch was compared in cases where methanol (**Figure S3a**) and deuterated methanol (**Figure S3b**) were used as the solvent.

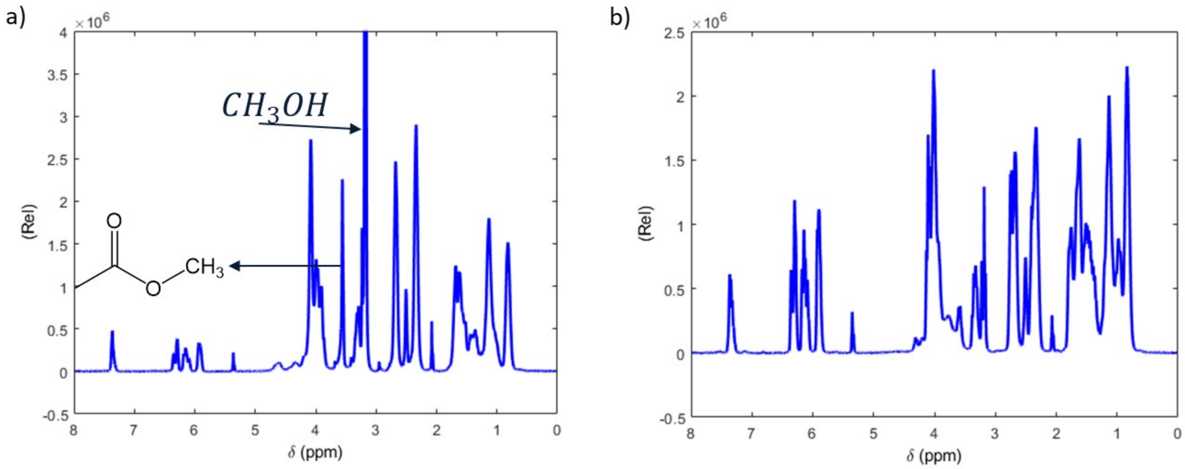

Figure S 3. *^{1}H NMR spectra of the polymerization batch (polymerization of cyclohexylamine and trimethylolpropane triacrylate) using methanol (a) and deuterated methanol (b) as the solvent.*

In the range between 3-4 ppm, the main differences between the two spectra is observed. In addition to the peaks of the methanol itself (3.2 and 4.0 ppm), there is an additional peak (at 3.55 ppm) which corresponds to the protons in the product of the transesterification reaction.

Because acrylates are prone to undergo such reactions, we have selected a triacrylamide, which is more stable towards reactions with alcohol and hydrolysis, instead of triacrylates for synthesis of hyperbranched polymers.

S3: Kinetics of aza-Michael addition of cyclohexylamine and triacrylamide

To compare the kinetics of aza-Michael addition reaction using acrylate and acrylamides, a kinetic investigation was conducted at RT, as shown in S4a.

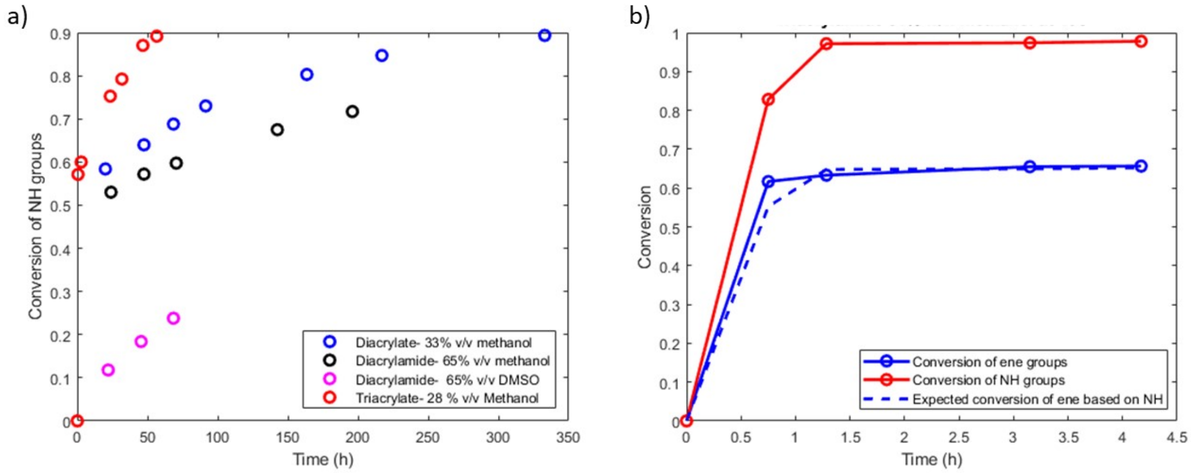

Figure S 4.*a) Conversion of NH groups in the aza-Michael addition at RT using triacrylate (red), diacrylate (blue), diacrylamide in methanol (black), and diacrylamide in dimethylsulfoxide (magenta), Conversion of the double bonds (blue) and the NH groups (red) in the polymerization of cyclohexylamine and 1,3,5-triacryloylhexahydro-1,3,5-triazine.*

In all the cases where methanol is used as the solvent, around 50% conversion is obtained relatively fast but the remaining NH groups react slower in case of diacrylamide compared to diacrylate as expected based on the reactivity of acrylates and acrylamides in aza-Michael addition reactions.[2] Variation of the solvent was also tested where dimethylsulfoxide (DMSO) was resulted in even slower reactions as observed in **Figure S4a**. For the reaction of triacrylamide, methanol was used as the solvent but a higher temperature (40 °C) was applied to improve the solubility of the reaction mixture and to enhance the reaction rate. **Figure S4b** displays the conversion of the aza-Michael addition in polymerization of cyclohexylamine and triacrylamide in a 1:1 ratio at 40 °C. The conversion of NH groups reached values higher than 90% after around 80 minutes at 40 °C indicating the efficiency of the synthesis method for polymerization. Polymers of varying molar mass distribution were obtained by varying polymerization times from around 4 h to around 8 h, the details of which are presented in Section S4.

S4: Molar mass investigation of the model system (polymer of cyclohexylamine and triacrylamide)

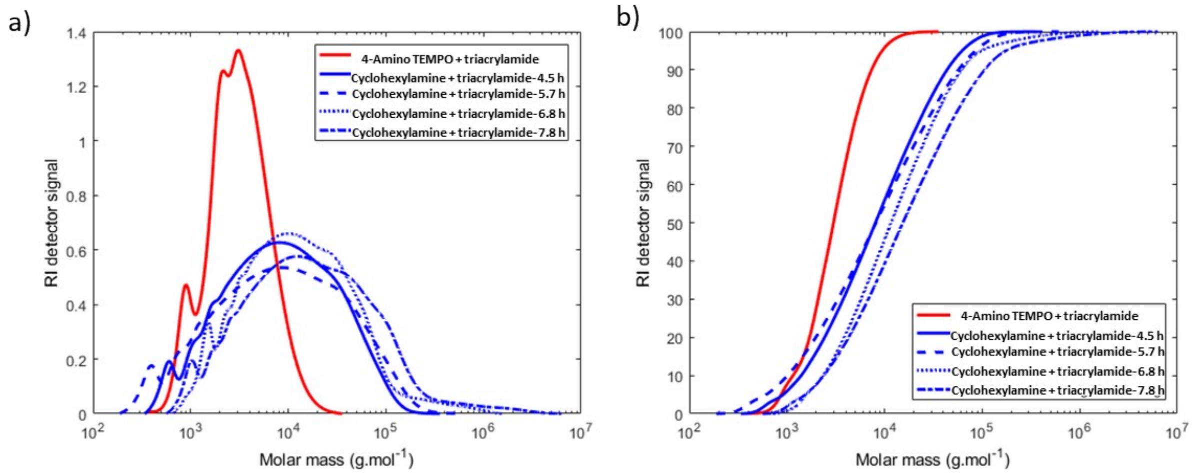

Figure S 5. *a) Molar mass distribution and b) cumulative molar mass distribution of polymers obtained from polymerization of cyclohexylamine and 1,3,5-triacryloylhexahydro-1,3,5-triazine at various polymerization times (blue) and the polymer obtained from polymerization of 4-amino TEMPO and 1,3,5-triacryloylhexahydro-1,3,5-triazine (red).*

Figure S5 shows the SEC-based molar mass distribution of the polymers obtained from polymerization of cyclohexylamine and triacrylamide. A broad molar mass distribution is observed which becomes broader with increasing polymerization time giving rise to an increasing fraction of large molar masses. In comparison, 4-amino TEMPO under the same polymerization condition produces polymers of relatively lower molar mass and narrower dispersity. Furthermore, with increasing polymerization time, the molar mass of the polymer with 4-amino TEMPO does not change significantly (data not shown).

S5: Structural characterization of hyperbranched TEMPO-based polymers

Figure S6 displays ^{1}H NMR spectra of HPT-I0 to HPT-I3 in D_2O after reduction of TEMPO with phenylhydrazine. While most of the protons appear to be in the range of 2 to 3 ppm, the presence of both monomers is indicated by the peak at 1.07 corresponding to the protons of the methyl groups of the TEMPO moiety and the peak at 5.24 corresponding to the protons of the triazine ring. In case where 1-methylpiperazine is involved in the structure the protons of that moiety should also appear in the range of 2 to 3 ppm.

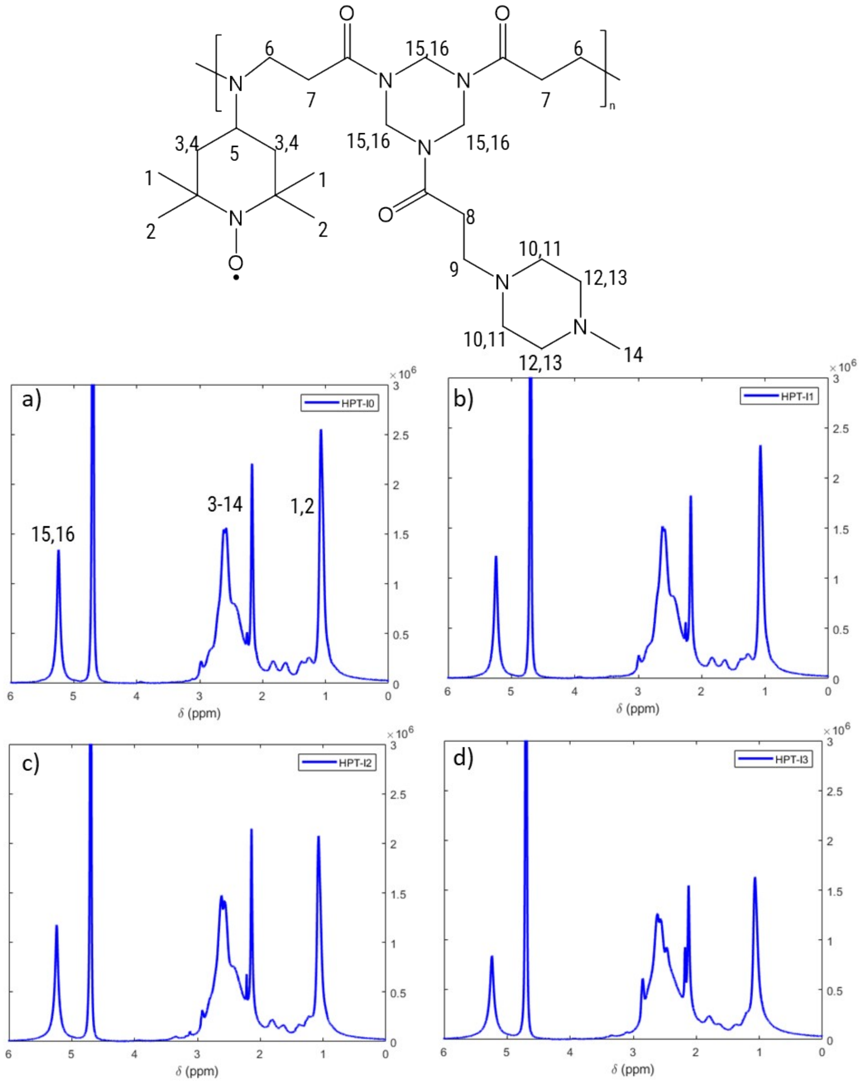

Figure S 6. *^{1}H NMR spectra (300 MHz, D_2O) of HPT-I0 (a), HPT-I1 (b), HPT-I2 (c), and HPT-I3 (d): 5.24 (s,6H, $H_{15,16}$), 2.17-2.98 (28H, H_{3-14}), and 1.07 (12H, $H_{15,16}$). The peak at 4.70 (s) belongs to the residual solvent in D_2O. In case of HPT-I1 to HPT-I3, the peaks due to the incorporation of 1-methylpiperazine should also appear in the range of 2 to 3 ppm.*

Furthermore, the presence of the TEMPO radical is also demonstrated by UV absorption spectroscopy. **Figure S7a** shows the absorption spectra of 4-amino TEMPO in the range of 200 to 400 nm for various concentrations. An absorption occurs in this range with a peak around 243 nm characteristic of the Π- Π* transition of TEMPO.[3] The spectra of the TEMPO-based polymers also reveals absorption at a similar range but in case of polymers the absorption is affected by more intense absorptions at lower wavelengths. Anyhow, at the same mass concentration of the polymers (0.08 mg mL^{-1}), the trend of absorption (HPT-I0> HPT-I1> HPT-I2~HPT-I3) agrees with the trend of TEMPO incorporation in the hyperbranched polymers.

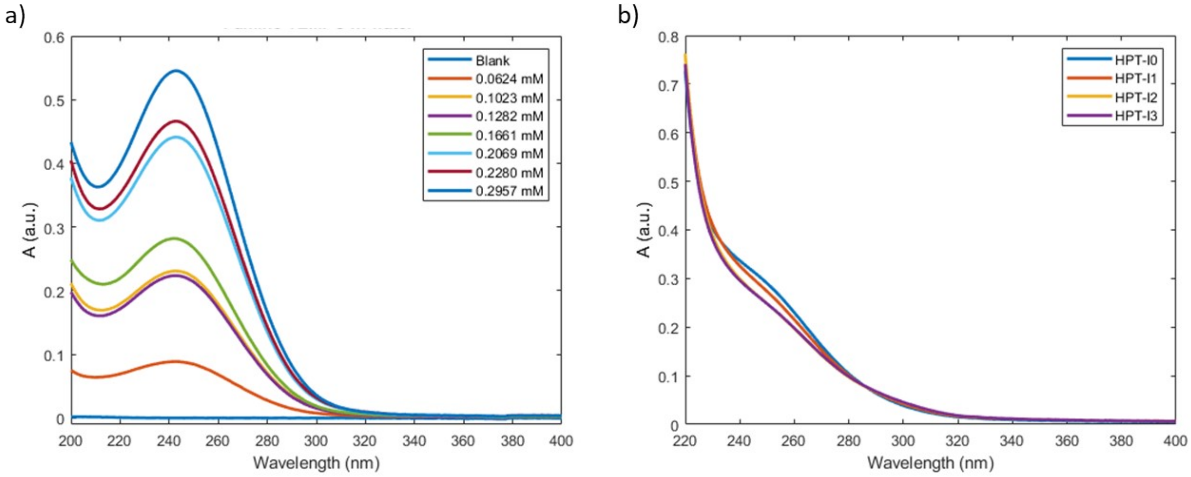

__Figure S 7.__ UV-Vis absorption spectra of 4-amino TEMPO (a) and hyperbranched polymers (b) in water.

S6: Electrochemical interference of tertiary amines and I^-

After the methylation reaction using iodomethane the counterions of the resulting polyelectrolyte are iodide. Iodide can be oxidized within the potential window of the TEMPO oxidation interfering with the electrochemical response of the polymer and affecting the battery performance. Therefore, it is important to substitute I^- by Cl^- which is electrochemically inactive in the desired potential window and also more hydrophilic compared to I^-. To systematically investigate the effect of residual I^-, we tested the model polymer system in which cyclohexylamine was used instead of 4-amino TEMPO and, thus, beside I^- no additional electrochemical response was expected. The first trial of ion exchange was conducted using ion exchange resins. **Figure S8** displays the cyclic voltammogram of a polymer model system before and after the methylation reaction and after various times of ion exchange processes. While before methylation no significant peak is observed around 0.6 V and 0.8 V *versus* Ag/AgCl reference electrode, such peaks appear after methylation using iodomethane. However, with substitution of I^- by Cl^-, the electrochemical response is declined. Compared to free I^- in the system (NaI salt in 1 M NaCl solution) the oxidation potential of the polymer counterions are shifted to higher potentials which can be due to the binding of I^- to the immobilized charges on the polymer which stabilize the reduced form and requires a higher potential for oxidation.

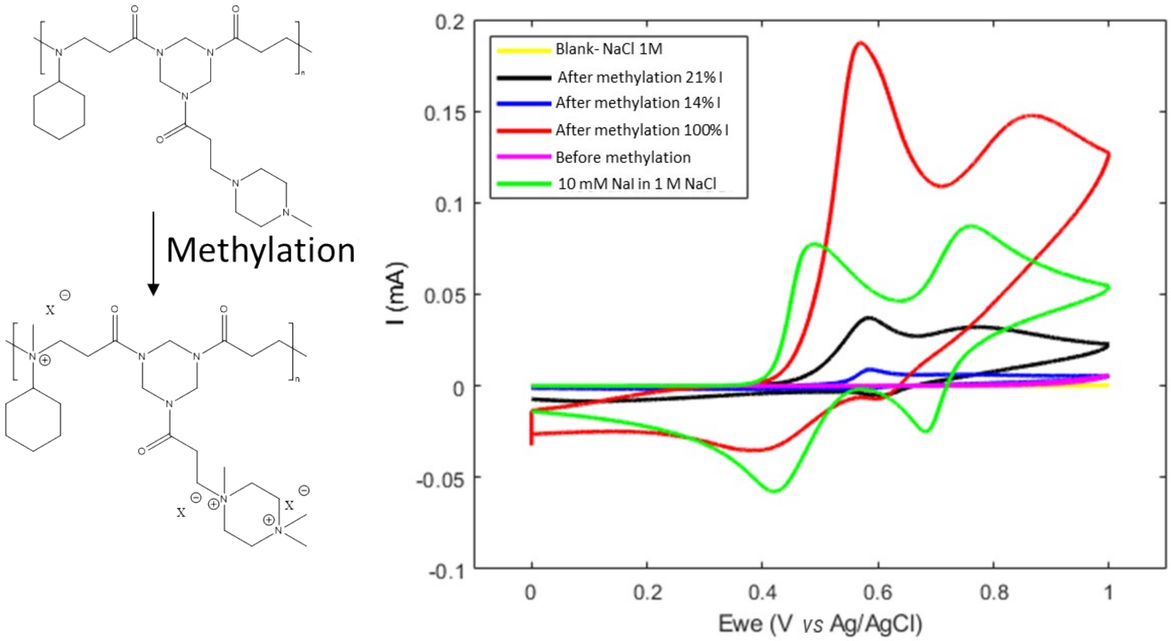

__Figure S 8.__ Cyclic voltammograms of the model polymer before and after methylation and ion exchange. The electrochemical oxidation is due to the presence of iodide ions in the system. The percentage of iodide ions are obtained from elemental analysis.

Furthermore, the electrochemical influence of tertiary amines was investigated through comparison of voltammograms of the model polymer system under basic and acidic conditions. When the pH value of the solution was not adjusted, the solution was basic (pH = 9.9) due to the presence of basic tertiary amine

groups. The voltammogram of that case is shown in **Figure S9** (magenta-solid line). Compared to the blank electrolyte, a rise in current is observed starting at around 0.7 V *versus* Ag/AgCl. Nevertheless, within the potential window of this experiment, no peak current is observed for this process. We attribute this behavior to the oxidation of the amine to a cation radical.[4] Under acidic conditions (pH value adjusted to 1.8), the tertiary amines are expected to be protonated and the voltammogram (magenta-dashed line) overlaps with that of the blank electrolyte indicating no electrochemical activity within the studied potential window.

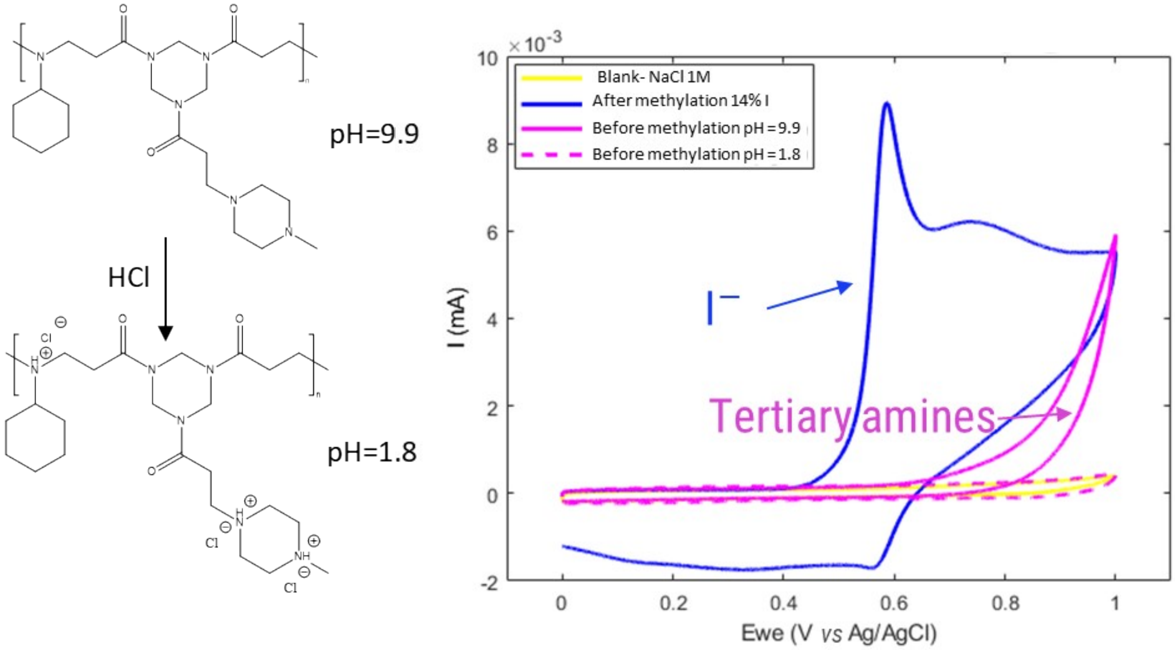

Figure S 9. *Cyclic voltammograms of the model polymer before methylation at basic (pH = 9.9) and acidic (pH = 1.8) conditions.*

Based on the electrochemical activity of I^-, we have conducted cyclic voltammetry (CV) on the polymers after ion exchange to determine any residual amount of I^- present in the system. Voltammograms of the TEMPO-based polymers after ion exchange using ion exchange resin and dialysis are shown in **Figure S10**. While ion exchange using the ion exchange resins with an excess amount (mass ratio of 20:1 relative to polymer mass) did not result in a complete removal of I^- (indicated by the peak around 0.6 V *versus* Ag/AgCl in the blue voltammogram and 3% remaining I^- based on elemental analysis), dialysis with an excess amount of 1 M NaCl solution was proven effective as no such peak is present in the product after ion exchange process using dialysis (red curve). The voltammogram of small molecule *N,N,N*-2,2,6,6-heptamethylpiperidinyloxy-4-ammonium chloride (TMATEMPO, black curve) is provided for comparison.

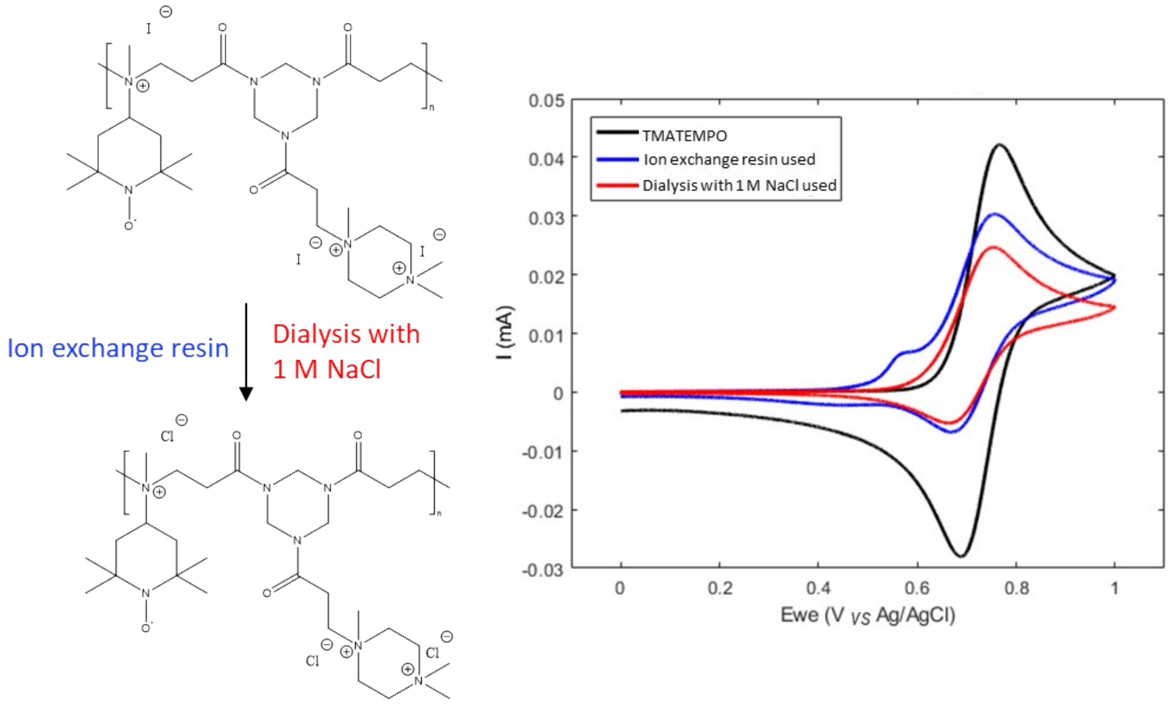

Figure S 10. *Voltammograms of TEMPO-based polymers at 100 mV s*$^{-1}$ *after ion exchange using ion exchange resin (blue) and using dialysis with 1 M NaCl solution (red). The black curve belongs to TMATEMPO for comparison.*

S7: Verification of the methylation reaction

To verify the post-polymerization reaction, HPT-I3 is selected because HPT-I3 contains the most amount of the trifunctional amine which corresponds to the highest number of tertiary amines present in the molecules among other samples. The methylation reaction was verified qualitatively by comparing the ^{1}H NMR spectra of the polymer before and after methylation. As shown in **Figure S11**, the peaks of the protons next to the tertiary amine groups which appear in the range of 2 to 3 ppm shift to higher δ (around 2.7 to 4 ppm) after methylation reaction indicating amine quaternization as previously reported.[5]

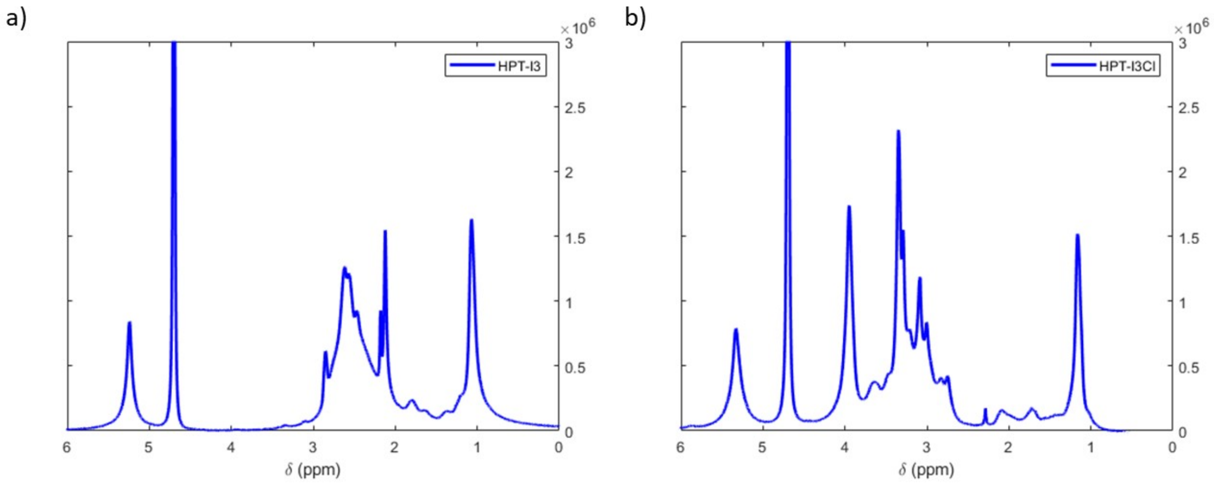

Figure S 11. *^{1}H NMR spectra of HPT-I3 before (a) and after (b) methylation. By quaternization of the tertiary amine groups through the methylation reaction, the peak of protons next to the ammonium group shifts to higher δ from the range of 2 to 3 ppm to 2.7 to 4 ppm.*

As a quantitative way of determining the conversion of the methylation reaction, we have used titration. Tertiary amine groups are basic in contrast to quaternary ammonium groups. Therefore, from a simple acid titration, the amount of residual basic groups and corresponding conversion of the methylation reaction could be obtained. Polymer solutions of HPT-I3 and HPT-I3Cl with equivalent number of repeating units (104 mg HPT-I3 and 136 mg HPT-I3Cl) were prepared in 1.5 M NaCl and the pH value of the polymer solutions were monitored with the controlled addition of a 0.1 M HCl in 1.5 M NaCl solution. The amounts of the solutions were adjusted so that if no base was present in the system, 0.1 mL of the added 0.1 M HCl would decrease the pH value of the solution to 3.0. **Figure S12** shows that while HPT-I3 required 5.6 mL of the added solution to consume the basic groups, HPT-I3Cl required only 0.4 mL. from the ratio of these values a conversion of 93 % was calculated for the methylation reaction.

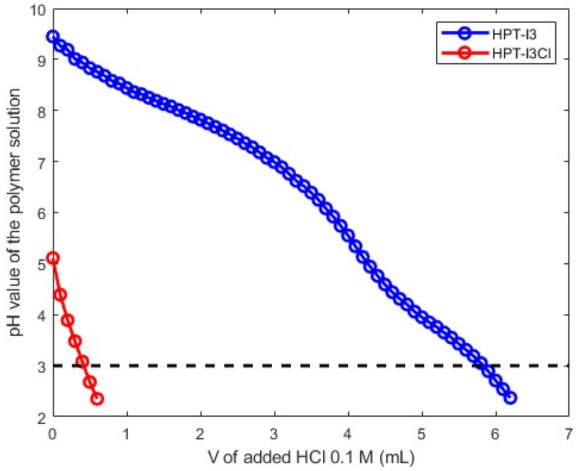

Figure S 12. *pH value of the polymer solutions as a function of added HCl solution. From the amount of required acid solution for each polymer the conversion of the methylation reaction is estimated to be around 93%.*

Finally, the conversion of the methylation reaction can also be estimated based on the amount of iodide in the system after the reaction. For HPT-I0, the exact mass of the repeating unit before and after methylation are 520.7 and 946.5 g mol^{-1}, if the methylation reaction is 100%. This corresponds to an iodide content of 40.2% w/w. The conversion of the methylation reaction can be estimated by equation S1 and S2:

$$\frac{X \times (126.9)}{520.7\ + X \times (141.9)} = \%\, I \qquad \text{(S1)}$$

$$Conversion = \frac{X}{3} \qquad \text{(S2)}$$

Table S 1. *Iodide content of the polymers after methylation reaction based on elemental analysis.*

Polymer sample	HPT-I0	HPT-I1	HPT-I2	HPT-I3
Iodide content after methylation reaction (% w/w)	39.4	38.7	40.2	41.4

From the equations S1 and S2 and based on the experimentally determined iodide content of HPT-I0 after methylation, a conversion of 96 % is estimated for the methylation reaction of HPT-I0. However, because of the incorporation of the trifunctional amine (1-(2-aminoethyl) piperazine, AEP) in case of HPT-I1, HPT-I2, and HPT-I3, the exact repeating unit of these polymers is unknown and, thus, equations S1 and S2 do not apply accurately.

S8: Hydrodynamic characterization using analytical ultracentrifugation (AUC)

The analysis of the experimentally obtained sedimentation velocity AUC data with the $c(s)$ model results in two parameters of the systems – the sedimentation coefficient, s ,and the translational frictional ratio, f/f_{sph}. The diffusion coefficient, D, under conditions of sedimentation is defined by

$$D = \frac{kT(1 - \upsilon\rho_0)^{1/2}}{\eta_0^{3/2} 9\pi\sqrt{2}\,(f/f_{sph})^{3/2}(s\upsilon)^{1/2}} \qquad \text{(S3)}$$

where k is Boltzmann constant, T is the temperature, υ is the partial specific volume, η_0 and ρ_0 are solvent viscosity and density, respectively.

The intrinsic sedimentation coefficient, $[s]$, and the intrinsic diffusion coefficient, $[D]$, are defined as

$$[s] = \frac{s\eta_0}{(1 - \upsilon\rho_0)} \qquad \text{(S4)}$$

$$[D] = \frac{D\eta_0}{T} \qquad \text{(S5)}$$

Knowing these intrinsic properties together with the intrinsic viscosity, $[\eta]$, one can calculate the hydrodynamic invariant A_0:

$$A_0 = \left(R[s][D]^2[\eta]\right)^{\frac{1}{3}} \qquad \text{(S6)}$$

If the value of the molar mass is known, $[D]$ can be substituted resulting in the following expression:

$$A_0 = R\,[s][\eta]^{\frac{1}{3}}M^{-\frac{2}{3}} \qquad \text{(S7)}$$

A_0 is known to indicate self-consistency of experimentally determined characteristics of the system and can indicate an adequacy of molar mass determinations.[6] Depending on polymer conformation, the values of A_0 can vary. Values below the expected range would indicate overestimation of the molar mass of the polymers. It appears that SEC coupled with MALLS detector tends to overestimate molar masses, except perhaps for HPT-I3. At the same time, AUC analysis results in molar masses that likely tend to be underestimated.

Table S2. *Molar mass and hydrodynamic properties of the hyperbranched polymers determined by AUC.*

$Sample$	$[\eta]$ (cm^3 g^{-1})	υ (cm^3 g^{-1})	s (S)	f/f_{sph}	D (10^{-6} cm^2 s^{-1})
HPT-I0	4.8	0.74	0.39	1.23	2.07
HPT-I1	6.3	0.73	0.55	1.21	1.81
HPT-I2	6.1	0.72	0.84	1.14	1.63
HPT-I3	10.2	0.73	2.08	1.11	1.06

Table S3. *Comparison of the molar masses and hydrodynamic invariants obtained via SEC and AUC*

$Sample$	$M_{W,\ SEC}$ (g mol^{-1})	$A_{0,SEC}$ (10^{-10} g cm^2 s^{-2} K^{-1} mol$^{-1/3}$)	$M_{s,f}$ (g mol^{-1})	A_0 (10^{-10} g cm^2 s^{-2} K^{-1} mol$^{-1/3}$)
HPT-I0	3,100	1.4	1,100	3.5
HPT-I1	4,100	1.7	1,700	3.8
HPT-I2	7,600	1.7	2,900	4.0
HPT-I3	18,300	2.5	11,300	4.8

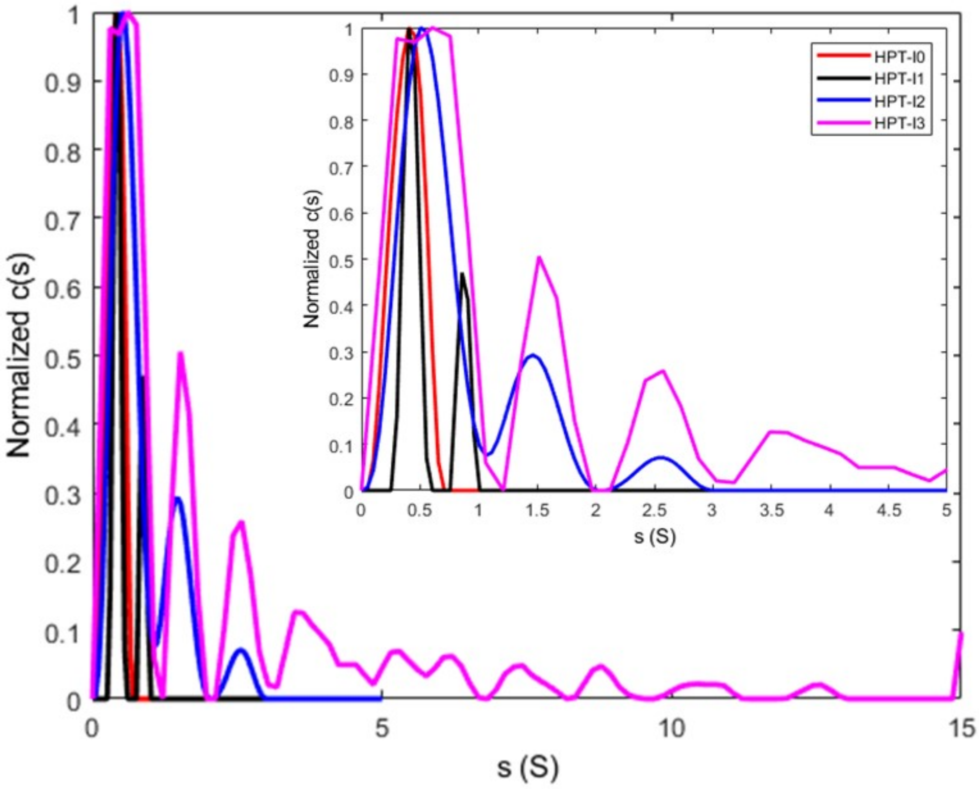

Figure S 13. *Normalized differential distribution of sedimentation coefficients based on sedimentation diffusion analysis c(s).*

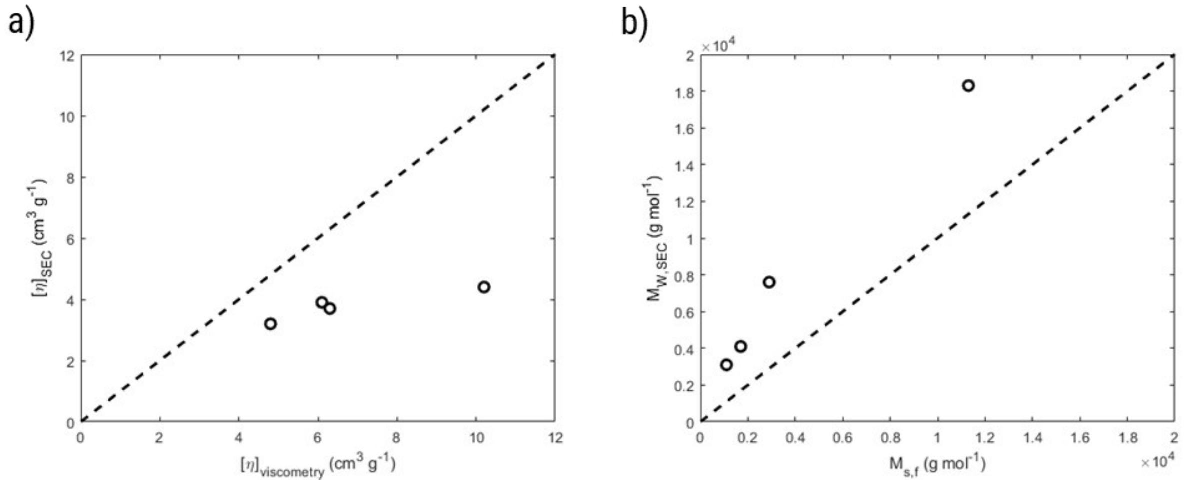

Figure S 14. *a) Comparison of intrinsic viscosities,* $[\eta]$*, obtained from SEC with viscometric detection (y-axis) and that of obtained from viscometry using the capillary ball combination (x-axis). b) Molar mass obtained from SEC (y-axis) plotted against the one obtained from AUC (x-axis).*

S9: Synthesis route for preparation of HPT-II1Cl

Scheme S2 presents the synthesis route for HPT-II1Cl. In this sample the polymerization conditions were similar to HPT-I1 but the termination was performed using 4-amino TEMPO instead of 1-methylpiperazine to incorporate more redox active moieties in the polymer chains. The post-polymerization modification was performed under similar conditions to those of HPT-I0 to HPT-I3. However, it should be noted that since the resulting polymer contains a secondary amine, the methylation reaction might have converted it to a tertiary amine but not all the way to quaternary ammonium. If complete quaternization is aimed, addition of a base is suggested. However, in this work, we have not added an additional base to prevent possible side reactions, *e.g.,* possible cleavage of the amide groups, TEMPO undergoing side reactions, etc., that can potentially occur under basic conditions. Instead to ensure that any residual unquaternized group would not affect the electrochemical properties, the electrochemical tests were performed at pH = 2.0 where the tertiary amines are protonated. The ion exchange process in this case was continued until no trace of iodide was detected *via* CV.

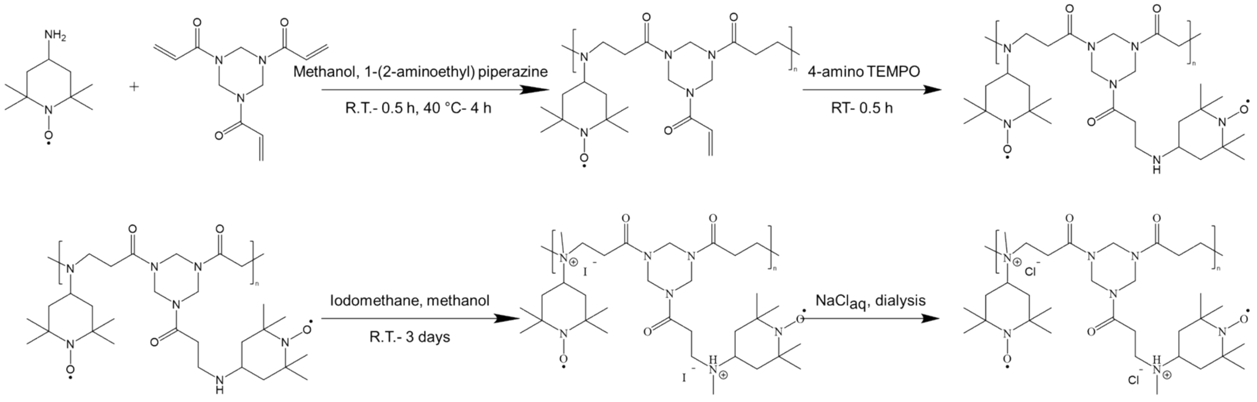

Scheme S 2. *Schematic representation of the polymerization of 4-amino TEMPO and 1,3,5-triacryloylhexahydro-1,3,5-triazine and termination using 4-amino TEMPO (top). Post-polymerization modification of polymers through amine quaternization and ion exchange (bottom).*

S10: Calculation of the polymer mass equivalent to 1 mole active TEMPO

The mass of the polymers that are equivalent to 1 mole of TEMPO was calculated based on the repeating units of the polymer. In the case where 1-(2-aminoethyl) piperazine (AEP) was not used, *i.e.* HPT-I0, the repeating unit is known. Assuming a complete quaternization and ion exchange process the molar mass of the resulting repeating unit is 672.2 g mol^{-1}. In other cases that AEP is involved, the molar mass is obtained as follows. Since in the polymerization the limiting functionality is the acrylamide group, the number of moieties involved in the polymer is calculated based on 1 mole of triacrylamide (3 moles double bond). Each mole of 4-amino TEMPO is considered to have 2 moles of reactive NH groups and, thus, reacting with 2 moles of double bonds and each mole of AEP is considered to have 3 moles of reactive NH groups. The remaining double bonds are consumed in termination either by 1-methylpiperazine (in case of HPT-I1, HPT-I2, and HPT-I3) or by another 4-amino TEMPO (in case of HPT-II1) both with 1 reactive NH group. It is assumed that all the amines become methylated and the counterion exchange is complete. To clarify, the calculations for HPT-I2Cl is provided as an example:

To consume 1 mole of triacrylamide, 3 moles of reactive NH is required. 2 moles come from the 4-amino TEMPO, and $3 \times 0.2 = 0.6$ moles come from AEP (considering the molar ratio of 0.2:1 relative to 4-amino TEMPO in case of HPT-I2), then the remaining ($3 - (2 + 0.6) = 0.4$) comes from 1-methylpiperazine. Therefore, 1 mole repeating unit contains in average: 1 mole triacrylamide, 1 mole 4-amino TEMPO (with 1 mole additional CH_3Cl for each mole after quaternization and ion exchange), 0.2 mole AEP (with 3 moles additional CH_3Cl for each mole AEP), and 0.4 mole 1-methylpiperazine (with 2 moles additional CH_3Cl for each mole AEP). The resulting equivalent mass to 1 mole TEMPO for HPT-I2 becomes

$$1 \times (249.27) + 1 \times (171.26 + 1 \times 50.49) + 0.2 \times (129.20 + 3 \times 50.49) + 0.4 \times (100.16 + 2 \times 50.49) = 607.61\ g.mol^{-1}$$

.

Table S4 summarizes the values of the calculated and experimentally determined (using electron paramagnetic resonance spectroscopy, EPR) mass of the polymers equivalent to 1 mole TEMPO.

***Table S 4**. Calculated and EPR-based mass of the polymers equivalent to 1 mole TEMPO moiety.*

Polymer sample	HPT-I0Cl	HPT-I1Cl	HPT-I2Cl	HPT-I3Cl	HPT-II1Cl
Calculated mass equivalent to 1 mole TEMPO (g mol^{-1})	672	640	608	575	385
Experimental mass equivalent to 1 mole TEMPO (g mol^{-1})	1,050	1,100	1,160	1,390	610

S11: Electrochemical properties of HPT-I2Cl and HPT-I3Cl

Figures S15 and **S16** display the results of CV and chronoamperometry (CA) of the HPT-I2Cl sample. From these data, diffusion coefficients of D = 1.1 × 10^{-6} cm^2 s^{-1} and D = 1.5 × 10^{-6} cm^2 s^{-1} are obtained for HPT-I2Cl based on CV and CA, respectively.

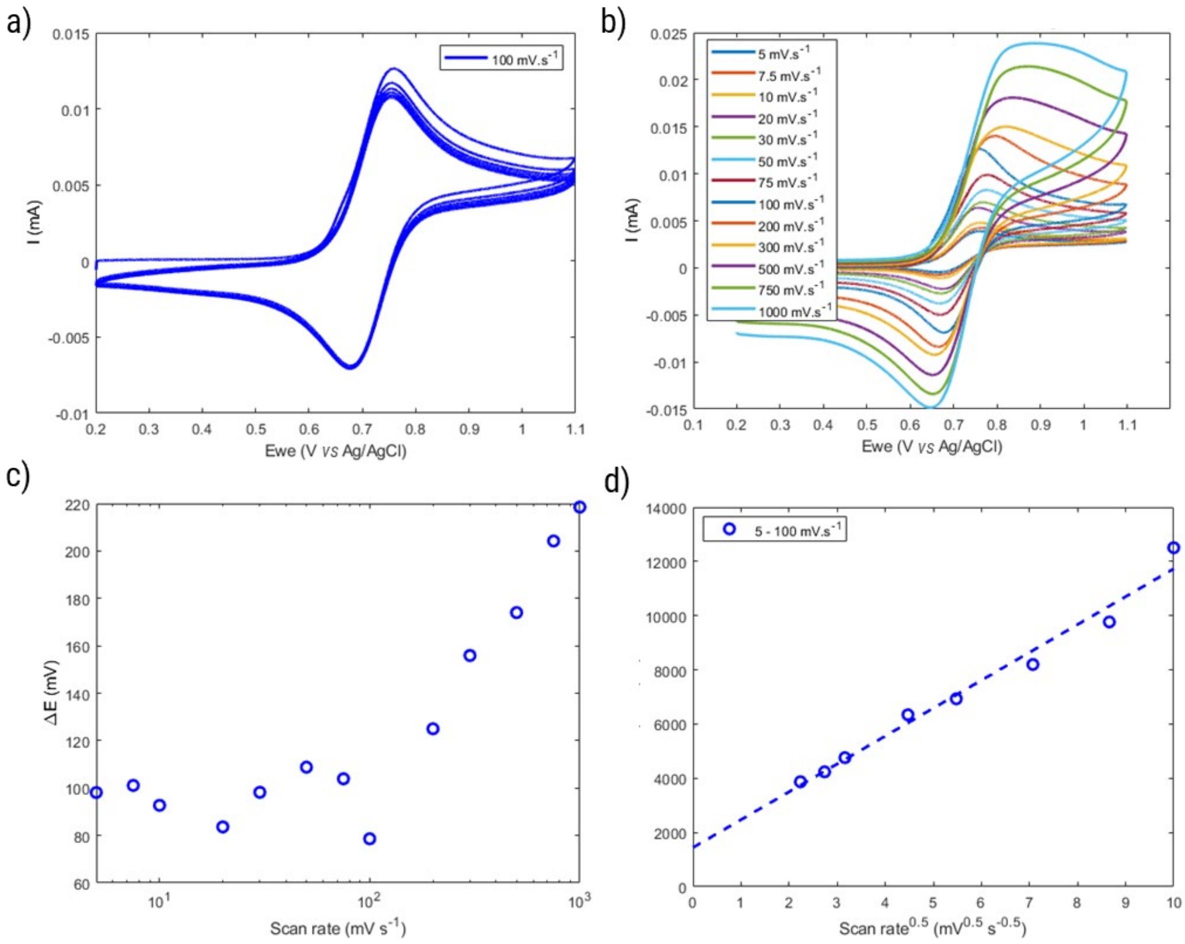

Figure S 15. *a) Cyclic voltammogram of HPT-I2Cl (5.7 mM active TEMPO) in 1.5 M NaCl aqueous solution at pH = 2.0 and at the scan rate of 100 mV* s^{-1} *(six consecutive cycles). b) First cycle voltammograms at various scan rates. c) Peak-to-peak potential difference (obtained from the first cycle voltammograms) as a function of scan rate. d) Oxidation peak current as a function of square root of scan rate in the range of scan rates of 5 to 100 mV* s^{-1}*.*

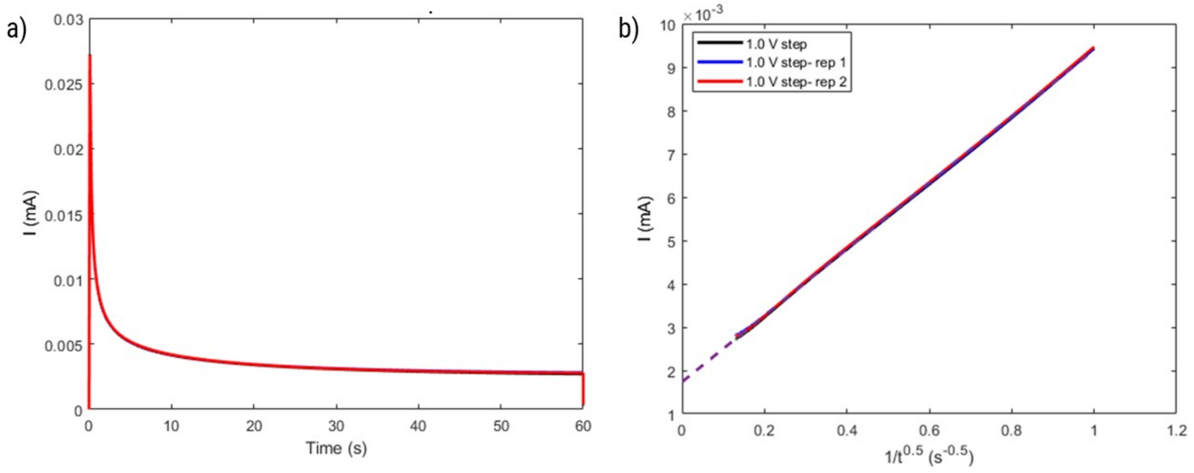

Figure S 16. *a) Current as a function of time and b) current as a function of inverse square root of time obtained from a step-potential chronoamperometry test on HPT-I2Cl (5.7 mM active TEMPO) in 1.5 M NaCl solution at pH = 2.0.*

Figures S17 and **S18** show the results of CV and CA on HPT-I3Cl sample. From these data, diffusion coefficients of D = 1.1 $\times$ 10^{-6} cm^2 s^{-1} and D = 1.2 $\times$ 10^{-6} cm^2 s^{-1} are obtained for HPT-I3Cl based on CV and CA, respectively.

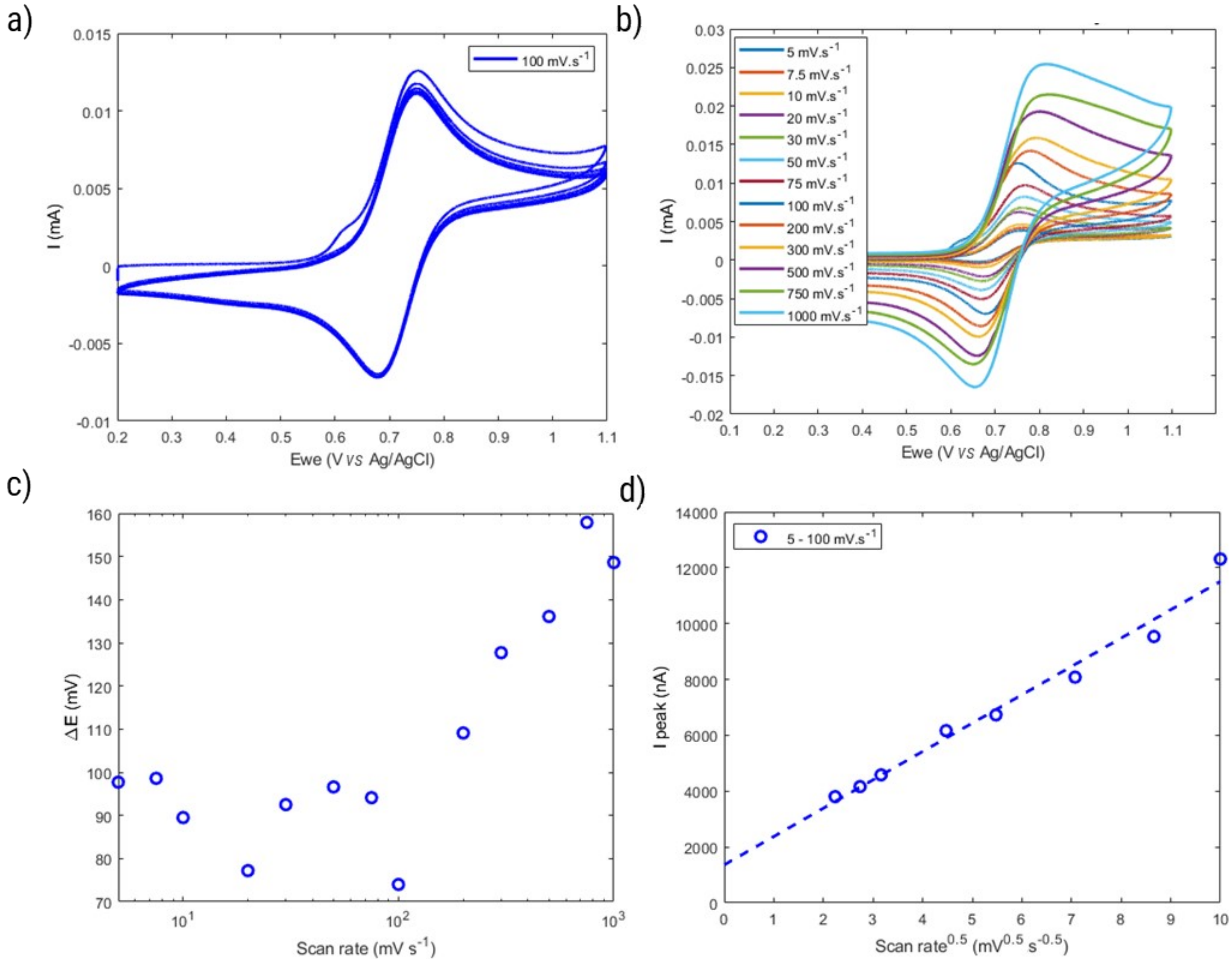

Figure S 17. *a) Cyclic voltammogram of HPT-I3Cl (5.7 mM active TEMPO) in 1.5 M NaCl aqueous solution at pH = 2.0 and at the scan rate of 100 mV s^{-1} (six consecutive cycles). b) First cycle voltammograms at various scan rates. c) Peak-to-peak potential difference (obtained from the first cycle voltammograms) as a function of scan rate. d) Oxidation peak current as a function of square root of scan rate in the range of scan rates of 5 to 100 mV. s^{-1}.*

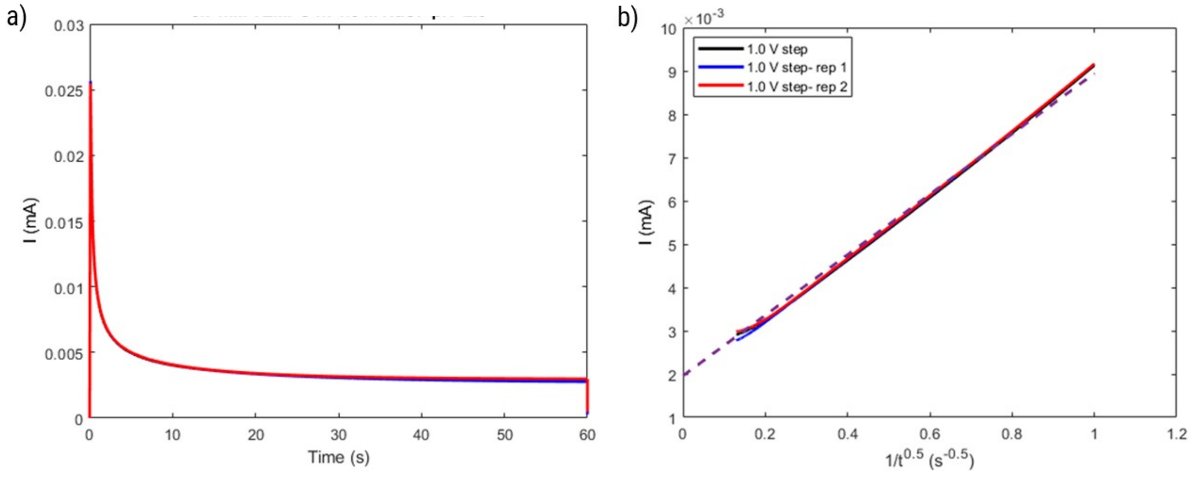

Figure S 18. *a) Current as a function of time and b) current as a function of inverse square root of time obtained from a step-potential chronoamperometry test on HPT-I3Cl (5.7 mM active TEMPO) in 1.5 M NaCl solution at pH = 2.0.*

S12: Angular dependence of the viscosity data

The viscosity of polymer solutions can be shear-rate dependent. To study the shear rate dependency, we have conducted viscometric measurements on a selected polymer solution at various angles corresponding to different shear rates. As reported in **Table S5**, solution viscosity of HPT-I3Cl which is the highest molar mass sample, show an insignificant dependency on the measurement angle.

Table S 5. *Viscosity of HPT-I3Cl in 1.5 M NaCl solution at angles from 20 ° up to 80 ° at a temperature of 20 °C.*

$C\ (g\ dl^{-1})$	$\eta_{dyn}\ (mPa\ s)$						
	20	30	40	50	60	70	80
38.2274	-	-	-	59.945	-	-	-
29.9182	22.188	21.693	22.135	22.042	21.871	21.705	21.4680
24.9451	12.632	12.827	12.846	12.853	-	-	-
20.10475	7.5634	7.7058	7.7318	7.7388	7.7571	7.7989	-
14.96066	4.6063	4.6835	4.6527	4.6269	4.6294	4.6615	4.7127
11.99123	3.3327	3.3942	3.4113	3.4099	3.4228	3.4249	3.4412
9.992477	2.8001	2.8331	2.8355	2.835	2.8539	2.8715	2.9035
7.983114	2.3211	2.3653	2.3716	2.3732	2.3764	2.3872	2.3815
5.970246	1.9563	1.9887	1.996	1.9967	2.0013	1.9964	1.9956
3.98856	1.6616	1.6854	1.6917	1.6916	1.6924	1.7008	1.7035
1.989315	1.4121	1.4338	1.436	1.4337	1.4359	1.4412	1.4399

S13: Further analysis of the battery performance

One of the possible causes of the deactivation of TEMPO moieties was considered to be side reactions of iodide during the methylation reaction. To investigate this possibility a polymer was synthesized similar to HPT-II1 but the methylation reaction was performed using chloromethane instead of iodomethane. The reaction was conducted by exposure of a solution of 5 g of polymer in 40 mL of methanol to chloromethane gas in a 50 mL reactor at room temperature. The reaction progress was monitored through consumption of the chloromethane in the gas phase. At the beginning a quick pressure drop was observed but with further consumption the pressure decrease slowed down. To maximize the reaction yield, the reactor was left under a pressure of around 3 bar which decreased to around 2 bar overnight. After precipitating the polymer product in diethyl ether, it was dissolved in water and dialyzed overnight using a dialysis membrane with MWCO of 1000 Da followed by lyophilization. The resulting polymer revealed a mass equivalent to 1 mole TEMPO of 557 ± 33 g mol^{-1} which was slightly lower than that of HPT-II1Cl (608 ± 27 g mol^{-1}) determined by EPR.

The battery performance of the resulting polymer (HPT-II1ClM) was investigated through an RFB experiment under similar conditions to that of HPT-II1Cl, as shown in Figure S19. This time before cycling a sample of the resulting catholyte solution was taken (around 0.7 mL) as a reference sample to compare with the sample after cycling through *ex-situ* characterization. Thus, the theoretical capacity of this battery (4.6 mA h) was slightly lower than that of HPT-II1Cl (5 mA h). In the first cycle, overcharging was observed followed by a discharge to only about 20% of the theoretical capacity. The capacity of the battery revealed a fast decay in the next cycles. This behavior was similar to that of HPT-II1Cl and, thus, it was concluded that replacing the iodomethane by chloromethane did not improve the performance of the polymer under battery conditions. The possible side reactions of iodide were not the cause of the overcharging and fast capacity decay under battery conditions.

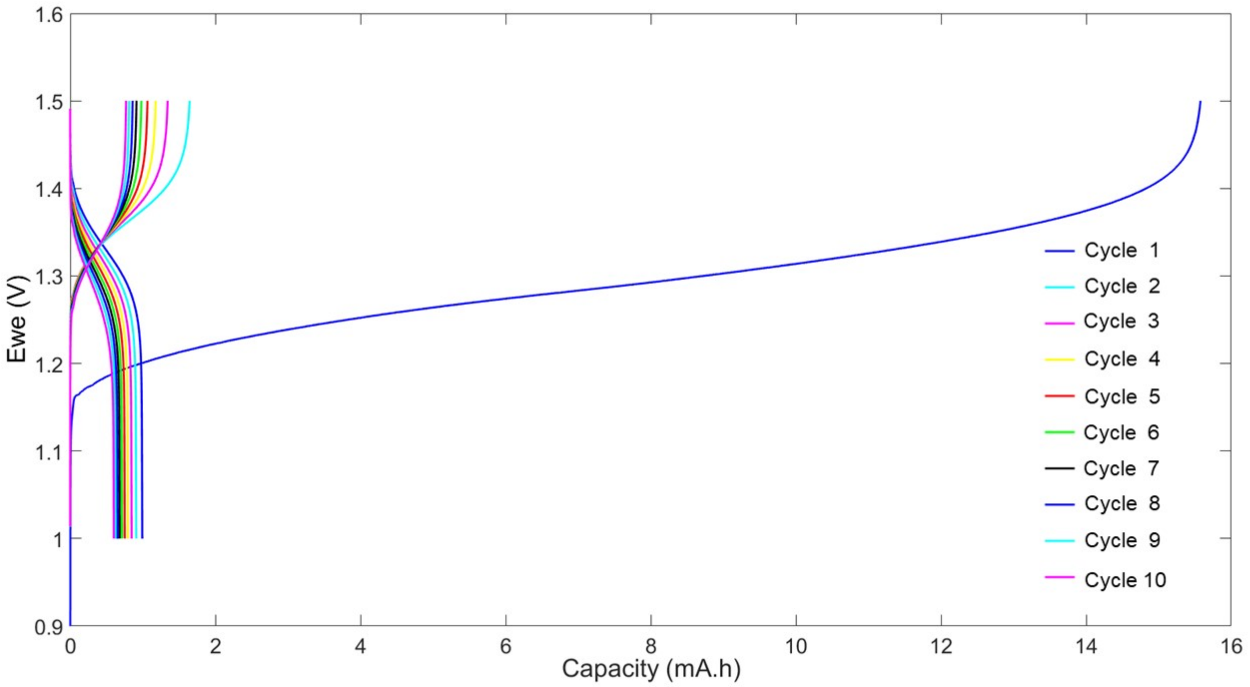

Figure S 19. Cell potential and capacity during the first 10 cycles of galvanostatic charging and discharging using 9.3 mL solution of the polymer methylated with chloromethane (HPT-II1ClM) (18.65 mM active TEMPO in 1.5 M NaCl solution at pH = 2.0 corresponding to theoretical capacity of 4.6 mA h) as the catholyte and 25 mL solution of MV (18.65 mM in 1.5 M NaCl solution at pH = 2.0) as the anolyte at a current of 5 mA.

The cycling was continued overnight after which a sample was obtained to compare with the reference sample before cycling. First the pH value of the obtained catholyte solution was measured which was still pH = 2.0 after cycling indicating that either the hydrogen evolution had not occurred on the anolyte side or the amount of protonium consumption on the anolyte side had not been significant enough to change the pH value of the catholyte solution and, thus, the remaining tertiary amine groups of the polymer are expected to stay protonated during cycling. To determine the presence of TEMPO, the resulting solutions were diluted with the same electrolyte solution used in the battery (1.5 M NaCl at pH=2.0) to reach a concentration of 1 mM for which cyclic voltammetry was performed, as shown in Figure S20.

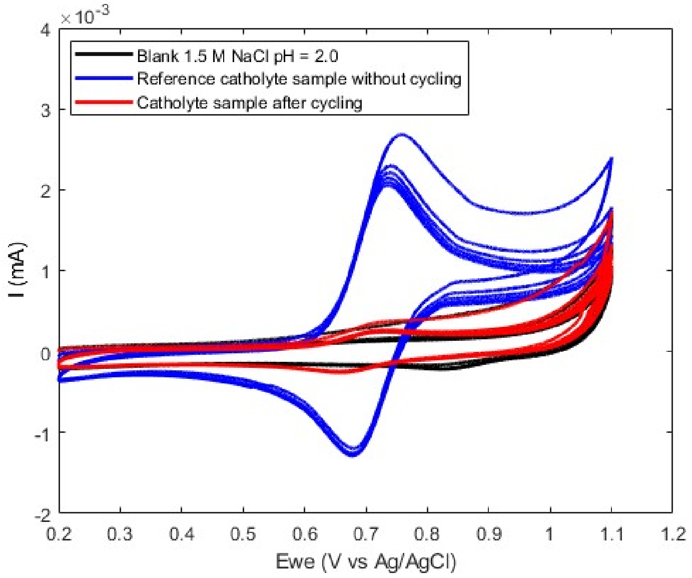

Figure S 20. Cyclic voltammograms of the diluted catholyte solutions (1 mM) without cycling (blue), after cycling (red), and the blank electrolyte solution (1.5 M NaCl at pH = 2.0, black).

The catholyte sample after cycling revealed insignificant peaks for oxidation and reduction processes indicating that TEMPO moieties were mostly degraded during cycling while a reference catholyte sample without cycling revealed significant redox activity corresponding to active TEMPO moieties. It can be concluded that disproportionation of the TEMPO moieties in the acidic electrolyte solution cannot solely explain the TEMPO degradation as degradation was mainly affected by cycling.

S14: Redox potential of other TEMPO derivatives for comparison

Table S6. The formal redox potential of previously reported TEMPO-based small molecules and polymers in comparison with that of this work.

Chemical structure	Formal potential	Reference	Comparison to HPT-II1Cl
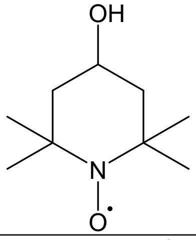	0.8 V (*vs.* SHE)	7	< $E^0_{HPT\text{-}II1Cl}$
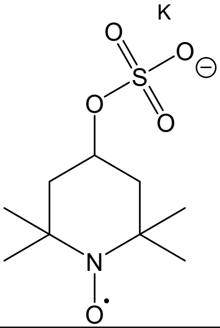	0.61 V (*vs.* Ag/AgCl)	8	< $E^0_{HPT\text{-}II1Cl}$
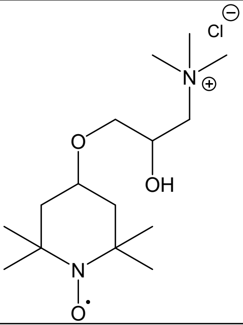	0.57 V (*vs.* Ag)	9	< $E^0_{HPT\text{-}II1Cl}$
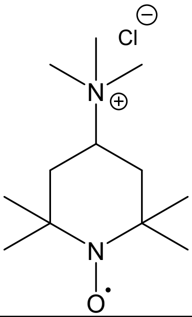	0.79 V (*vs.* Ag/AgCl)	10	> $E^0_{HPT\text{-}II1Cl}$

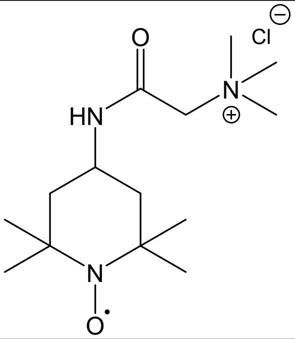	0.64 V (*vs.* Ag/AgCl)	11	< $E^0_{HPT\text{-}II1Cl}$
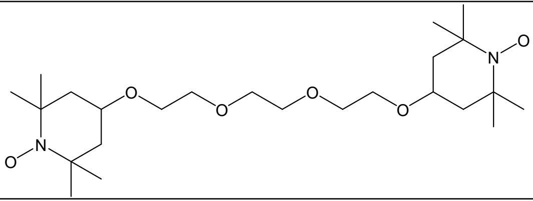	0.28 V (*vs.* Fc/Fc^+)	12	< $E^0_{HPT\text{-}II1Cl}$
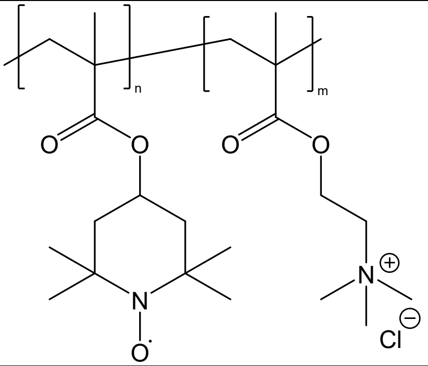	0.7 V (*vs.* Ag/AgCl)	13	< $E^0_{HPT\text{-}II1Cl}$
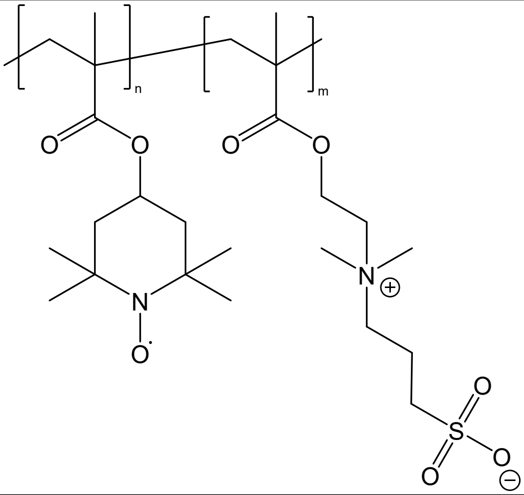	0.7 V (*vs.* Ag/AgCl)	14	< $E^0_{HPT\text{-}II1Cl}$

S15: Diffusion coefficient and charge transfer rate constant of TEMPO-based polymers for comparison

*Table S 7. The diffusion coefficient and the electron transfer rate constant of previously reported TEMPO-based polymers and that of this work. *Nonaqueous electrolyte solutions.*

Chemical structure	D (cm^2 s^{-1})	k^0 (cm s^{-1})	Reference
	1.0 × 10^{-6}	1.9 × 10^{-3}	This work
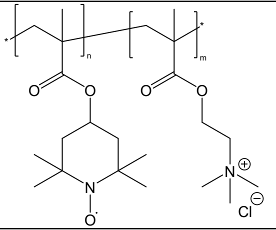	7.0 × 10^{-8}	4.5 × 10^{-4}	13
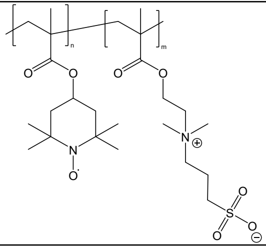	3.8 × 10^{-7}	9.7 × 10^{-4}	14
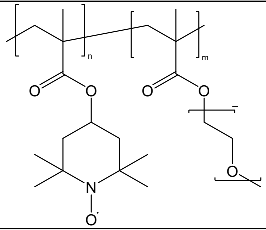	1.65 × 10^{-7}	9.9 × 10^{-4}	15 *
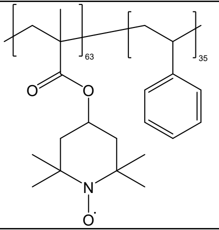	1.8 × 10^{-7}	7.0 × 10^{-4}	16 *
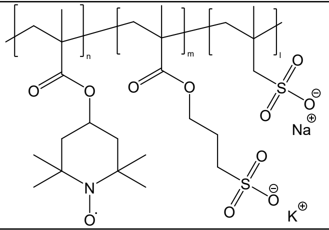	2.4 × 10^{-8}	7.8 × 10^{-4}	17

S16: Theoretical and actual capacity of TEMPO-based polymer catholytes for comparison

Table S 8. Theoretical capacity and actual capacity of TEMPO-based polymers used as catholytes in redox flow batteries.

Chemical structure	Theoretical capacity (Ah L^{-1})	Actual capacity (Ah L^{-1})	Reference
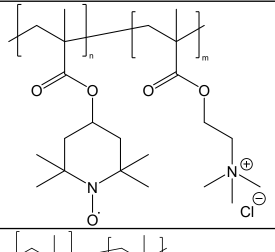	10	8 (at a current density of 40 mA cm^{-2})	13
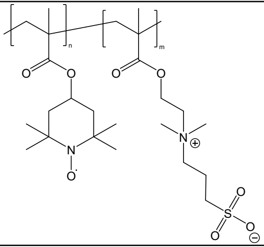	10	9 (at a current density of 8 mA cm^{-2})	14
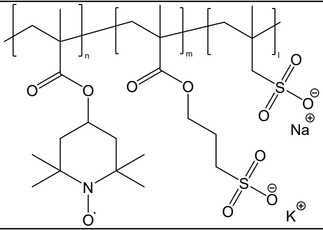	12	9 (at a current density of 10 mA cm^{-2})	17
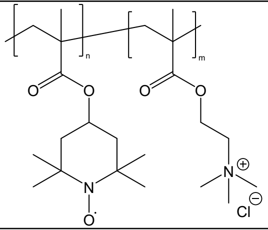	6.7	4.4	18

References

1. D. Wu, Y. Liu, C. He, Chung and Goh, *Macromolecules*, 2004, **37**, 6763-6770.
2. A. Narayanan, B. Maiti and P. De, *Reactive and Functional Polymers*, 2015, **91**, 35-42.
3. H. Fan, B. Hu, H. Li, M. Ravivarma, Y. Feng and J. Song, *Angewandte Chemie International Edition*, 2022, **61**, e202115908.
4. R. S. Deinhammer, M. Ho, J. W. Anderegg and M. D. Porter, *Langmuir*, 1994, **10**, 1306-1313.
5. V. Bütün, S. P. Armes and N. C. Billingham, *Macromolecules*, 2001, **34**, 1148-1159.
6. M. Grube, G. Cinar, U. S. Schubert and I. Nischang, *Polymers*, 2020, **12**, 277.
7. T. Liu, X. Wei, Z. Nie, V. Sprenkle and W. Wang, *Adv. Energy Mater.*, 2016, **6**, 1501449.
8. J. Winsberg, C. Stolze, A. Schwenke, S. Muench, M. D. Hager and U. S. Schubert, *ACS Energy Lett.*, 2017, **2**, 2, 411-416.
9. Z. Chang, D. Henkensmeier and R. Chen, *ChemSusChem*, 2017, **10**, 16, 3193-3197.
10. T. Janoschka, N. Martin, M. D. Hager and U. S. Schubert, *Angew. Chem. Int. Ed.*, 2016, **55**, 14427-14430.
11. P. Rohland, O. Nolte, K. Schreyer, H. Görls, M. D. Hager and U. S. Schubert, *Mater. Adv.*, 2022, **3**, 4278-4288.
12. T. A. Weiss, G. Fan, B. J. Neyhouse, E. B. Moore, A. Furst and F. R. Brushett, *Batteries & Supercaps*, 2023, **6**, 8, e202300034.
13. T. Janoschka, N. Martin, U. Martin, C. Friebe, S. Morgenstern, H. Hiller, M. D. Hager and U. S. Schubert, *Nature*, 2015, **527**, 78-81.
14. T. Hagemann, M. Strumpf, E. Schröter, C. Stolze, M. Grube, I. Nischang, M. D. Hager and U. S. Schubert, *Chemistry of Materials*, 2019, **31**, 7987-7999.
15. J. Winsberg, T. Janoschka, S. Morgenstern, T. Hagemann, S. Muench, G. Hauffman, J. F. Gohy, M. D. Hager and U. S. Schubert, *Adv. Mater.*, 2016, **28**, 2238-2243.
16. J. Winsberg, S. Muench, T. Hagemann, S. Morgenstern, T. Janoschka, M. Billing, F. H. Schacher, G. Hauffman, J.-F. Gohy and S. Hoeppener, *Polymer Chemistry*, 2016, **7**, 1711-1718.
17. H. Fu, C. Zhang, H. Wang, B. Du, J. Nie, J. Xu and L. Chen, *Journal of Power Sources*, 2022, **545**, 231905.
18. T. Hagemann, J. Winsberg, M. Grube, I. Nischang, T. Janoschka, N. Martin, M. D. Hager and U. S. Schubert, *Journal of Power Sources*, 2018, **378**, 546-554.
